# Near-Merger and Contextual Sensitivity in the Perception of /n-l/ in Sichuan Mandarin

**DOI:** 10.3390/brainsci16020155

**Published:** 2026-01-29

**Authors:** Minghao Zheng, Allen Shamsi, Ratree Wayland

**Affiliations:** Department of Linguistics, University of Florida, Gainesville, FL 32611-5454, USA; minghao.zheng@ufl.edu (M.Z.); allenshamsiev@ufl.edu (A.S.)

**Keywords:** Sichuan Mandarin, /n-l/, speech perception, vowel context effects, acoustic cue availability, near-merger

## Abstract

**Highlights:**

**What was done?**

- Sichuan Mandarin listeners’ perception of the /n-l/ contrast was examined using graded acoustic continua in different vowel contexts.
- Signal-detection and mixed-effects modeling were used to separate sensitivity, bias, and contextual cue effects.

**What was found?**

- The /n-l/ contrast patterns as a context-dependent near-merger, with substantially greater perceptual separation in /i/ than in /a/.
- Vowel-dependent acoustic cue structure, rather than decision bias, drives this asymmetry, showing how contrast representations depend on available phonetic evidence.

**Abstract:**

**Background/Objectives**: Sichuan Mandarin is often described as exhibiting overlap or merger between word-initial /n/ and /l/, but perceptual sensitivity across phonetic contexts remains underexplored. This study examines whether perception of the /n-l/ contrast varies by vowel context and listener experience. **Methods**: Thirty-two Sichuan Mandarin listeners completed categorical identification and same–different AX discrimination tasks using seven-step /n/ → /l/ continua derived from native-speaker productions in /i/ and /a/ contexts. Sensitivity, response bias, accuracy, and response times were analyzed alongside individual differences. Acoustic properties of the stimuli were quantified using spectral and amplitude-based measures. **Results**: Listeners showed overall reduced sensitivity to the /n-l/ contrast, with substantially stronger perceptual differentiation in /i/ than in /a/ contexts. Bias patterns were comparable across contexts, indicating sensitivity-driven effects. Acoustic analyses showed more robust cue structure in the /i/ continuum. Age, education, and Standard Mandarin experience modulated response efficiency but did not eliminate the vowel asymmetry. **Conclusions**: Results support a context-dependent near-merger of /n/ and /l/, shaped by acoustic cue availability and experience-based cue exploitation.

## 1. Introduction

Phonological mergers arise when a distinction between two or more phonological categories is reduced or neutralized, often following extensive overlap in their phonetic realizations [1]. The merger or alternation of word-initial nasal /n/ and lateral /l/ consonants has been documented in several Chinese languages and dialects, including Fuzhou Min [2], Cantonese [3], Nanjing Mandarin [4], Wuhan Mandarin [5], and Sichuan Mandarin [6]. While production data from these varieties demonstrate substantial overlap between /n/ and /l/, an open question is whether such patterns reflect complete phonological merger or intermediate states in which perceptual distinctions persist under specific phonetic or sociolinguistic conditions.

This question is particularly pertinent for speakers of regional Mandarin varieties who are also exposed to Standard Mandarin (Putonghua), where /n/ and /l/ remain phonemically distinct, with well-established acoustic and perceptual differentiation [2,3]. Exposure to Standard Mandarin introduces a stable contrast that may coexist with merger-like patterns in the regional variety, potentially yielding competing perceptual representations of the same segmental contrast. Sichuan Mandarin, a Southwestern Mandarin variety spoken by over 270 million speakers [6], therefore provides an informative testing ground for examining how dialectal variation and language contact shape perceptual representations of phonological contrast.

The present study investigates the perceptual differentiation of the /n-l/ contrast in Sichuan Mandarin using same–different AX discrimination and forced-choice identification tasks. Listeners’ sensitivity to the contrast was examined across vowel contexts and as a function of social factors, including age, gender, education, and Standard Mandarin proficiency and use. Additionally, we analyze perceptual judgments in relation to the acoustic structure of the stimuli, allowing assessment of whether /n-l/ alternation in Sichuan Mandarin patterns as a context-dependent near-merger, how gradient the perceptual representations are, and how sociolinguistic experience shapes contrast stability. The results speak to broader accounts of sound change by highlighting how phonetic context and language experience jointly constrain perceptual contrast maintenance.

## 2. Background

### 2.1. /n/ and /l/ Alternations in Sichuan Mandarin

Sichuan Mandarin, also known as Sichuanese, is a branch of Southwestern Mandarin spoken primarily in Sichuan and Chongqing and adjacent regions [7]. A widely noted characteristic of Sichuan Mandarin concerns the phonemic status of the coronal sonorant consonants /n/ and /l/. Descriptive accounts have long reported alternations between these segments in word-initial position, raising questions about whether they reflect free phonetic variation [8,9], directional neutralization [10], or a broader process of phonological merger [6,11]. Importantly, Sichuan Mandarin shares the same syllable structure as Standard Mandarin, in which /n/ and /l/ form a robust segmental contrast. Because Standard Mandarin serves as the medium of instruction throughout Sichuan Province, speakers are extensively exposed to a system that maintains a robust /n-l/ contrast. This sociolinguistic context makes Sichuan Mandarin a particularly informative case for examining how dialectal phonological patterns interact with perceptual representations of contrast, and whether reported /n-l/ alternations correspond to categorical merger or to context-dependent, gradient reorganization of phonetic cues.

Despite long-standing descriptive attention, there remains debate regarding the phonemic status of /n/ and /l/ in Sichuan Mandarin and the nature of their surface realizations. Early descriptive work suggested that syllable-initial /n/ may surface as an alveolar nasal [n], an alveolar lateral approximant [l], or a nasalized lateral approximant [$\tilde{l}$] [8]. Subsequent studies described variation primarily between [n] and [l] [9,12], with evidence further suggesting that tokens transcribed as [n] may in fact involve lateral airflow with nasalization [12]. The precise phonetic nature and distribution of these realizations remain unsettled, in part due to the limited availability of systematic acoustic or articulatory evidence.

Several studies have attempted to identify phonological or phonetic conditioning factors underlying the /n-l/ alternation. The Dialect Investigation Group [8] reported that [n] occurred more frequently before high front vowels such as /i/ and /y/, whereas [n], [l], and [$\tilde{l}$] appeared to be in free variation before other vowels. In contrast, Ma and Tan [9] described [n] and [l] as freely varying without specifying clear conditioning environments, and the status of the nasalized lateral approximant [$\tilde{l}$] was not systematically examined. These divergent accounts leave unresolved whether vowel context plays a consistent or robust role in shaping the /n-l/ pattern in Sichuan Mandarin.

Phonetic studies have likewise yielded mixed results. Shi [11] reported that Chengdu speakers—a representative variety of Sichuan Mandarin—produced both [n] and [l]-like variants with relatively high degrees of nasality. Mean nasalance was ~93.5% for nasal initials overall and ~61% for tokens in the merged /n~l/ category. Shi further reported systematic differences by vowel context, with higher nasalance in syllables containing high-front vocalic elements (e.g., /i, y/, or palatal-glide environments) than in syllables with non-high/front nuclei (e.g., /a, o, u/). Nasalance is a quantitative acoustic index of nasal resonance reported as a percentage and is interpreted as a gradient measure rather than a categorical diagnostic of phonological nasalization. In contrast, a study of Beijing Mandarin—a variety that maintains a robust /n-l/ contrast—reported a large nasality difference between /n/ (mean nasalance ≈ 91%) and /l/ (mean ≈ 32%; i.e., well below the non-nasal critical value of ~40). These findings show that nasalance is gradient and that even non-nasal sonorants may show substantial non-zero nasalance [13]. Taken together, these findings point to substantial phonetic overlap between realizations associated with /n/ and /l/ in Sichuan Mandarin, but they do not establish whether this overlap reflects free phonetic variation, a directional merger, or a phonological merger with residual phonetic structure.

Evidence for a directional or incomplete merger has been proposed on the basis of limited production data. In a single-case study of one Sichuan Mandarin speaker, Zhang [10] argued that the alternation reflects a one-way merger in which syllable-initial /n/ may be realized as [l], but not vice versa. However, the narrow empirical basis of this claim leaves its generality uncertain.

Zhang and Levis [6] further examined /n-l/ production by Southwestern Mandarin speakers in Standard Mandarin (L2) and English (L3). Although their study did not directly investigate L1 production, the results demonstrated persistent effects of speakers’ native phonological inventories on production in both additional languages. These findings suggest that merger-like patterns in Southwestern Mandarin are robust and not fully overridden by exposure to Standard Mandarin. At the same time, variability across speakers and contexts was observed, consistent with an incomplete or gradient merger rather than categorical contrast maintenance.

### 2.2. The Merger Between [l] and [n] in Other Chinese Languages

The /n-l/ merger is not unique to Sichuan Mandarin and has been documented in several other Chinese languages, providing useful comparative perspectives on the range of merger outcomes and the conditions under which perceptual sensitivity may be retained. One well-documented case comes from Fuzhou Min. Cheng et al. [2] reported converging production and perception evidence for a near-complete phonological merger of word-initial /n/ and /l/. Although speakers produced acoustically differentiated variants, listeners failed to reliably distinguish the two sounds, indicating a dissociation between production and perception. Importantly, younger Fuzhou Min listeners showed greater sensitivity to the contrast than older listeners, a pattern attributed to increased exposure to Standard Mandarin, which reintroduces the /n-l/ distinction into the linguistic environment.

Hong Kong Cantonese (HKC) presents a different developmental trajectory. In HKC, /l/ and /n/ have been described as occurring in complementary distribution, with [l] appearing in onset position and [n] in coda position [14]. However, this distribution has been progressively eroded by an ongoing merger, particularly among younger speakers [15,16]. Despite substantial overlap in production, Cantonese speakers retain some perceptual sensitivity to the /n-l/ distinction, indicating that the merger remains incomplete [17]. Taken together, evidence from Fuzhou Min and Hong Kong Cantonese demonstrates that /n-l/ mergers in Chinese languages vary in completeness and that sociolinguistic factors—most notably exposure to Standard Mandarin—can constrain their perceptual consequences.

In addition to cross-dialectal merger patterns, perceptual work on Standard Mandarin provides an important baseline for understanding vowel-context effects on the /n-l/ contrast in a non-merged system. Using speeded AX discrimination, Liu and Li [18] demonstrated that the /n-l/ contrast is perceptually more distinct in the low vowel context /a/ than in the high vowel context /i/, a pattern consistent with typological tendencies observed across Chinese dialects. More generally, research on nasality perception has shown that perceived nasalization depends on the interaction of multiple acoustic cues—particularly in the F1 region—and that large acoustic differences do not necessarily translate into proportional perceptual distinctiveness across vowel contexts [19].

Despite longstanding descriptive accounts of /n-l/ alternation in Sichuan Mandarin, relatively little is known about how this pattern is reflected in perception, particularly across phonetic contexts. Given the variability observed across Chinese languages and the documented role of Standard Mandarin exposure in shaping merger outcomes elsewhere, a perceptual investigation is necessary to determine whether residual sensitivity to the /n-l/ distinction persists in Sichuan Mandarin and whether such sensitivity is context-dependent. The present study addresses this gap by examining the perceptual discrimination of /n/ and /l/ across vowel contexts, informed by detailed acoustic analysis of the stimuli.

Using a combination of same–different AX discrimination and forced-choice identification tasks, the present study examines Sichuan Mandarin listeners’ sensitivity to the /n-l/ distinction across vowel contexts. In the identification task, we deliberately avoided the use of Chinese characters—unlike [2]—and instead employed picture-based responses to minimize potential orthographic and lexical biases. Although Chinese characters do not transparently encode segmental phonological form, they are strongly associated with conventionalized lexical pronunciations, particularly those of Standard Mandarin. Psycholinguistic research has shown that orthographic representations can be automatically activated during spoken-word recognition, biasing listeners toward top-down lexical expectations rather than fine-grained acoustic information [20]. In Chinese, character recognition reliably engages phonological representations despite the absence of systematic grapheme–phoneme correspondence, and these phonological activations are closely tied to standardized lexical pronunciations [21]. As a result, character-based response formats may encourage reliance on canonical lexical forms, potentially obscuring listeners’ sensitivity to gradient acoustic cues—especially for speakers of regional varieties whose phonological systems do not robustly maintain the Standard Mandarin /n-l/ contrast. Importantly, the use of picture-based responses does not assume intact phonological contrast or collapsed lexical representations. Even in systems exhibiting phonological merger or near-merger, speakers maintain distinct lexical entries for words historically containing /n/ versus /l/; perceptual difficulty arises from mapping acoustically ambiguous input onto these lexical categories rather than from the absence of lexical knowledge.

Despite extensive descriptive attention, existing work does not yet provide a clear account of how /n-l/ overlap in Sichuan Mandarin is represented perceptually. Prior production-based and nasalance-based findings suggest substantial phonetic overlap between /n/ and /l/ realizations, but the extent to which listeners maintain a perceptual distinction—and whether that distinction is context-dependent—remains unresolved. In particular, prior reports disagree on whether vowel context conditions /n-l/ variation, and phonetic evidence remains inconclusive as to whether overlap reflects free variation, a directional merger, or a phonological merger with residual phonetic structure.

A controlled perception study is therefore necessary to test (i) whether Sichuan Mandarin listeners show reliable sensitivity to the /n-l/ contrast under graded cue manipulation, (ii) whether such sensitivity differs by vowel context, and (iii) whether sociolinguistic experience with Standard Mandarin modulates sensitivity and/or response patterns.

Specifically, the study addresses the following research questions:

RQ1: To what extent do native speakers of Sichuan Mandarin perceptually differentiate /n/ and /l/, and does this differentiation vary across vowel contexts?

RQ2: How do social and experiential factors—such as age, gender, education, proficiency in Standard Mandarin, and language use—modulate perceptual differentiation between /n/ and /l/?

Based on prior descriptive work on Sichuan Mandarin and comparative evidence from other Chinese languages, we advance the following hypotheses. First, if the /n-l/ contrast is reduced or incompletely maintained in Sichuan Mandarin, listeners are expected to show limited or gradient perceptual differentiation between /n/ and /l/, rather than categorical contrast. Second, perceptual differentiation is expected to be stronger in the high vowel context /i/ than in the low vowel context /a/, reflecting vowel-dependent cue availability in the present stimuli. Finally, listeners with greater exposure to Standard Mandarin—particularly younger and more highly educated speakers—are expected to show weaker merger-like patterns, reflecting partial maintenance or reweighting of perceptual cues associated with the /n-l/ contrast.

## 3. Materials and Methods

### 3.1. Participants

Thirty-two adults from Deyang, China took part in the study (24 females, 8 males; age range 31–56 years). Female participants had a mean age of 42.58 years (SD = 7.17); males 47.75 years (SD = 7.01). Recruitment and testing occurred in a city mall during the Summer of 2025. All participants reported normal hearing and no history of speech or language disorders. Participation was voluntary and uncompensated.

All listeners were native speakers of Sichuan Mandarin (L1) and reported speaking Standard Mandarin (Putonghua) as an L2. Language background was assessed with the LEAP-Q [22]. Education averaged 10.73 years (SD = 2.48; median = 12; range = 5–16). Highest attained level: 1 professional training, 3 college graduates, 12 some college, 2 technical secondary school, 14 less than high school. Current language exposure (self-reported percentage of time) was L1: M = 85.8%, SD = 10.3, median = 90, min–max = 60–99; L2: M = 14.2%, SD = 10.3, median = 10, min–max = 1–40. Preferred language for speaking when the interlocutor is equally fluent: L1: M = 86.09%, SD = 14.85, median = 90, min–max = 50–100; L2: M = 13.91%, SD = 14.85, median = 10, min–max = 0–50.

No audiometric screening, vision/literacy requirements, or handedness criteria were imposed, and no a priori exclusion criteria were applied beyond native-language status. Consent materials were written in Standard Mandarin, and all study instructions were delivered in Sichuan Mandarin. The protocol was approved by the University of Florida IRB board, and all participants provided verbal informed consent.

### 3.2. Stimuli

**Figure 1:**
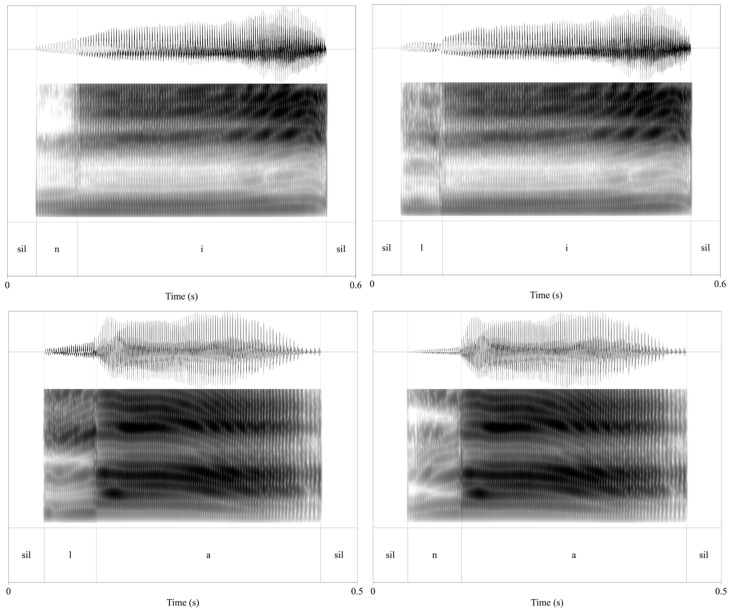
Waveform, spectrogram, and TextGrid annotations for the endpoint tokens /ni2/ ‘pear’ (**top left**) and /li2/ ‘mud’ (**top right**), as well as /na4/ ‘that’ (**bottom left**) and /la4/ ‘spicy’ (**bottom right**). All tokens include 50 ms silence padding at onset and offset. Vertical boundaries indicate segmentation of the consonant (C) and vowel (V) intervals used for stimulus construction and acoustic analysis.

We constructed two seven-step /n/ → /l/ continua (i.e., one for /a/ and one for /i/ vocalic context) from an unambiguous /n/ (step 0) to an unambiguous /l/ (step 6) (Figure 1). Waveform editing (silence padding, segmentation, duration normalization, and intensity normalization) was performed in Praat software (version 6.4.38 [23]), and only the spectral morphing and resynthesis of the consonant–vowel (CV) window were implemented using a custom Python (Version 3.9.12) script.

Endpoint tokens (/na4/ 那 ‘that’, /la4/ 辣 ‘spicy’, /ni2/ 泥 ‘mud’, /li2/ 梨 ‘pear’) were recorded at 44.1 kHz in a quiet hotel room (no sound attenuation) using the inline microphone of Huawei Classic In-ear Earphones (wired control) connected to a laptop running Praat software.

The talker was a 28-year-old female from Zhejiang Province, China, a native speaker of Standard Mandarin and a Wu variety, who holds a Mandarin secondary-level A certificate, which indicates high proficiency in Mandarin pronunciation. She did not exhibit an /n-l/ merger in the selected materials and was therefore well-suited to provide acoustically stable and consistently differentiated /n/ and /l/ endpoints for continuum construction. Because the goal of the present study is to assess Sichuan Mandarin listeners’ sensitivity to the phonetic cues supporting the /n-l/ contrast across vowel contexts (rather than to model Sichuan Mandarin production), using a talker who reliably maintains the contrast enables controlled manipulation of cue robustness while minimizing variability in endpoint productions. From 10 takes per item, endpoints were chosen on waveform criteria such that the consonant portion was ≥70 ms and the following vowel displayed steady F0 and formant structure. Each file was padded with 50 ms of silence at the beginning and end (prior to segmentation). A phonetically trained annotator set hand boundaries using zero-crossing marks, formant transitions, and intensity cues to isolate the consonant and initial-vowel coarticulation window and the remainder of the vowel. For tokens in /a/ context, tokens were duration-normalized to 400 ms of speech (plus the 50 ms pre-/post-silences); for /i/, tokens were normalized to 500 ms of speech (plus the silences). After resynthesis (see below), intensity normalization was performed in Praat, scaling the average intensity to 70 dB SPL and applying this uniform scaling across both vowel series and the practice items.

Continua were generated using a custom Python script that implements established methods for creating graded continua by linear interpolation of spectral-envelope amplitudes between two naturally produced endpoints [24,25,26]. Each continuum step was created by linearly interpolating the magnitude spectral envelope of the CV portion between the /n/ and /l/ endpoints on a frame-by-frame basis in the frequency domain, while holding phase, timing, and excitation constant. This procedure yields graded, multi-cue spectral variation across steps rather than perceptually equidistant phonetic increments. Specifically, for each continuum, a Short-Time Fourier Transform (STFT) was computed on the CV window (Hann window, 1024-point FFT, 75% overlap, hop size = 256 samples), yielding framewise magnitude spectral envelopes and phase information for analysis-synthesis [27]. To hold excitation source and temporal structure constant across steps, the /n/ endpoint was pre-emphasized (first-order high-pass filter, coefficient = 0.97), and its phase spectrum was reused for resynthesis at every continuum position.

Continuum steps were generated by linear framewise interpolation of the magnitude spectral envelopes of the /n/ and /l/ endpoints:

$$\alpha =\frac{s}{6},\;\;\;\;\;\;\;\;s\in \left\{0,\ldots ,6\right\};\;\;\;\;\;\;\;\;E_{\mathrm{step}\;s}=\left(1-\alpha \right)\;E_{/n/}+\alpha \;E_{/l/},$$

where $E_{/n/}\;$ and $E_{/l/}$ denote magnitude spectral envelopes of the endpoints, *s* the step index, and *α* the mixing weight. The interpolated envelope was combined with the fixed /n/-derived phase and converted back to the time domain via inverse STFT; reconstruction from modified STFT representations is well characterized in the signal-processing literature [28]. The morphed CV segment then replaced the original CV portion in the /nV/ token; the remainder of the vowel was left unchanged to preserve naturalness. Finally, files were peak-normalized to 99% prior to Praat-based intensity scaling. The final stimulus set comprised 14 tokens (2 vowels × 7 steps). Three practice items (/ba/ → /pa/; VOT = 13, 47, 87 ms) were used for task familiarization in both experiments (see Section 4 Procedures below).

Because the spectral-envelope interpolation causes multiple correlated acoustic cues (e.g., F2/F3 transitions, bandwidths, anti-resonances, spectral tilt) to shift jointly across steps, adjacent continuum steps are not expected to be equidistant in acoustic or perceptual space. Accordingly, the present study was not designed as a categorical perception experiment and did not assume equal spacing between continuum steps. Instead, the continua were intended to provide graded variation in the acoustic cues distinguishing /n/ and /l/, allowing us to assess listeners’ sensitivity to vowel-dependent cue availability and cue use rather than categorical boundary placement. Consistent with this design, step was treated as an ordered factor with successive-difference contrasts, without assuming equal perceptual spacing.

### 3.3. Acoustic Characteristics of the Stimuli

To characterize the acoustic properties of the /n-l/ continua and to assess how relevant cues varied across vowel contexts, we conducted acoustic analyses on all stimulus tokens. Following [2], we examined three measures previously shown to be informative for nasal–lateral contrasts: ΔA1, relative root mean square (RMS) amplitude, and first-formant bandwidth (BW1). ΔA1 was measured as the difference in the amplitude of the first-formant peak 20 ms after versus 20 ms before the consonant–vowel boundary. Relative RMS amplitude was computed as the difference in RMS amplitude (in dB) between the consonant interval and the following vowel interval, measured over their full durations. BW1 was estimated using Burg formant analysis as the bandwidth of F1 at 5, 10, 15, and 20 ms after vowel onset, with values averaged across these time points.

These measures are motivated by well-established acoustic consequences of nasal coupling. Prior work has shown that nasal segments and nasalized vowels exhibit attenuation of low-frequency oral energy, the introduction of low-frequency nasal resonances, and increased formant bandwidth due to added losses associated with coupling to the nasal tract [29,30]. In contrast, laterals are characterized by stronger oral resonance and more stable formant structure, yielding higher relative amplitude and narrower bandwidths. Accordingly, /n/ endpoints are expected to exhibit lower relative RMS amplitude, greater attenuation of A1 across the consonant–vowel boundary, and wider BW1 values relative to /l/ endpoints, though the magnitude of these effects may vary as a function of vowel context [2,30].

Although this implementation does not exactly replicate the VoiceSauce-based procedures used in prior work, it captures early-vowel spectral attenuation and broadening effects that have been shown to index nasalization and to contribute to nasal–lateral distinctions in both production and perception [2,29,30].

Table 1 summarizes descriptive acoustic characteristics of the seven-step /n-l/ continua in the /i/ and /a/ vowel contexts. Consonant and vowel durations were held approximately constant across continuum steps within each vowel context, ensuring that temporal properties were not systematically manipulated along the continua. Vowel duration differed across vowel contexts, with longer vowels in the /a/ series than in the /i/ series.

Across the acoustic dimensions examined, spectral and amplitude-based measures exhibited clear vowel-dependent scaling, consistent with prior work showing that the magnitude and recoverability of nasal-related acoustic cues vary systematically across vowel contexts [2,29,30]. Relative RMS amplitude varied systematically across continuum steps in both vowel contexts, with larger overall consonant–vowel amplitude differences in the /i/ continuum than in the /a/ continuum. ΔA1 showed a graded decrease from the /n/ endpoint (CV_0_) to the /l/ endpoint (CV_6_) in the /i/ continuum, whereas ΔA1 values in the /a/ continuum were substantially smaller and spanned a compressed range. In contrast, BW1 did not exhibit a monotonic change across continuum steps in either vowel context. BW1 values in the /a/ continuum remained highly stable across steps, while BW1 values in the /i/ continuum showed greater variability, including elevated values at later continuum positions. Taken together, these patterns indicate that while amplitude-based measures were systematically graded along the continua, BW1 was not uniformly controlled across steps, consistent with prior findings that bandwidth-based correlates of nasality exhibit greater variability and vowel dependence.

## 4. Procedures

Each experimental session followed a fixed sequence. First, participants completed the Language Experience and Proficiency Questionnaire [22], administered in an interview format. They then completed a picture-naming task designed to elicit productions of the target tokens (production data are not reported here). The perceptual component followed, consisting of a same–different AX discrimination task and a forced-choice identification task, administered in that order.

All perceptual tasks were administered on a PC laptop (Microsoft Surface Pro 8) using PsychoPy (version 2025.1.1 [31]) in a quiet office room within a furniture mall. Stimuli were presented over Bose Headphones 700 (model 794297-0300; Bose Corporation, Framingham, MA, USA). Laptop output volume was held constant at 70/100 across participants, although absolute sound pressure levels were not calibrated. Responses were collected via the laptop keyboard, and both response accuracy and reaction time were recorded. Participants were instructed to keep their index fingers on the response keys throughout the task, to respond as quickly and accurately as possible, and to make a single response per trial. Short breaks were provided between task blocks.

### 4.1. AX Discrimination (Same–Different)

Participants heard two tokens (A and X) and judged whether they were the same or different. Oral instructions were delivered in Sichuan Mandarin, while the same text was displayed on the screen. The full Chinese instructions appear in Supplementary Materials S1.1. The blue-labeled key mapped to “same”, and the red-labeled key to “different”. In our setup, these were ‘S’ and ‘L’, respectively. Each token included 50 ms of leading and trailing silence (see Section 3.2. Stimuli above). The inter-stimulus interval (ISI) was 750 ms (nominal 650 ms plus the appended silences), selected to encourage phonological-level judgments (cf. [2,32]). The inter-trial interval (ITI) was 550 ms (500 ms plus the leading silence of the next A). Responses were disabled until the offset of X. AX response times were measured from X-offset to keypress. Participants had 3 s to respond, and if no key was pressed, the next trial began automatically.

During the practice (task familiarization) block, participants first responded to two repetitions of 8 AX pairs (4 same, 4 different covering AA, BB, AB, BA). No feedback was given. The main task was designed similarly, with trials blocked by vowel (/i/ first, then /a/ for all participants). Within each vowel block, participants completed 6 repetitions of 20 pairs: 10 same (Δ = 0) and 10 different (Δ = 2), where Δ = 2 referred to nominal step separation along the seven-step continuum (e.g., 1–3, 2–4, 3–5, 4–6, 5–7, plus reverse orderings). In total, 240 trials (20 pairs × 6 reps × 2 vowels) were presented in pseudorandomized order. No additional randomization constraints were imposed, and no feedback was provided. The AX task lasted approximately 25 min.

### 4.2. Identification (Forced-Choice Labeling)

In each trial, participants heard a single stimulus and selected which lexical category it belonged to by choosing between two pictorial referents displayed on the screen. One referent corresponded to the word /li2/ ‘pear’, and the other to /ni2/ ‘mud’. The two referents were presented side by side and were not labeled with Chinese characters or phonetic transcriptions, in order to avoid orthographic or lexical bias.

Oral instructions were given in Sichuan Mandarin, and the matching on-screen text appears in Supplementary Materials S1.2. The blue-labeled button on the keyboard selected the left image (key ‘S’), and the red-labeled button selected the right image (key ‘L’). Side assignment of category images was randomized in each trial (e.g., /li/ “pear” vs. /ni/ “mud”). Images were generic, publicly available pictures (sourced via web search), chosen to be easily nameable. Luminance standardization was not critical for this task. The pictures appeared only after stimulus offset to discourage anticipatory responses.

The ITI matched AX (550 ms), matching the AX task. Trials were also blocked by vowel (/i/ then /a/). Participants first completed practice block (6 items × 2 repetitions) with no feedback, followed by 168 experimental trials. For each vowel, each of the seven continuum tokens was presented 12 times in total: six times with the two response labels displayed in one left–right order (e.g., the “pear” picture on the left and the “mud” picture on the right) and six times with the labels reversed, resulting in 7 tokens × 6 repetitions × 2 choice orders × 2 vowels = 168 trials. Participants had 3 s to respond, and if no key was pressed, the trial timed out and the program advanced. Identification RTs were measured from stimulus offset to keypress. The task lasted about 7 min.

Across the two perceptual tasks, each participant contributed dense repeated observations (240 AX trials and 168 identification trials). This design prioritizes within-participant contrasts and supports precise estimation of effect sizes and confidence intervals, while maintaining limited sensitivity to very small effects at N = 32.

### 4.3. Statistical Analysis

Although AX preceded the Identification task in each session, the Statistical Analysis and Results sections present Identification first to establish the category mapping and response bias that contextualize AX discrimination.

All analyses were run in R 4.3.1 [33]. Data import and wrangling used the tidyverse stack [34]. Graphics were produced with ggplot2 [35]. Mixed-effects logistic and linear models were fit with lme4 [36]; Satterthwaite-approximated *p*-values came from lmerTest [37]. Estimated marginal means and planned contrasts were obtained with emmeans [38] using Holm adjustment [39], and model checks used performance and DHARMa [40,41]. For factor coding, we used sum contrasts and successive-difference contrasts from MASS [42]; models were optimized with the BOBYQA algorithm [43]. Lastly, for SDT sensitivity indices, we used log-linear-corrected d′, drawing on a compact R routine by Christophe Pallier [44].

#### 4.3.1. Identification Task: Responses and Reaction Times

**Responses.** We modeled trial-level /l/ responses (1 = /l/, 0 = /n/) with a binomial generalized linear mixed model (GLMM; lme4, logit link). Primary fixed effects included Step (CV_0_–CV_6_), Vowel (/i/, /a/), and their interaction. Step was treated as an ordered factor and coded with successive-difference (adjacent) contrasts to target local changes from step *k* to *k* + 1, appropriate for ordinal continua that do not need to be perceptually equidistant. This allows non-linear identification functions without imposing a single slope. Vowel and Gender were sum-coded, yielding a grand-mean intercept. Continuous covariates (Age, Education Years, L2 Standard Mandarin Use: mean of LEAP-Q exposure and speaking %, and L2 Standard Mandarin Proficiency: mean of LEAP-Q speaking and understanding) were z-scored. Guided by a priori hypotheses [2], wherein older listeners, females, lower L2 use and proficiency, and lower education flatten identification slopes, and /i/ contexts yield steeper slopes, we specified Step × Vowel, Vowel × Age (baseline bias by vowel), and Step × {Age, Proficiency, Education, Gender} (as slope modulation). Random effects included by-subject intercepts and an uncorrelated by-subject slope for Vowel to capture individual response biases with stable convergence for a two-level slope.

Model selection followed a sequence of nested likelihood-ratio tests under maximum likelihood (Laplace). Fixed-effect inferences were based on Wald z statistics and likelihood-ratio tests. Evidence consistent with a merger was defined as flattened adjacent-step contrasts within a vowel (odds ratios ≈ 1) and/or convergence of the /i/ and /a/ functions across steps. Planned comparisons (emmeans) targeted (a) adjacent-step contrasts within each vowel (and, where moderators were of interest, at −1/0/+1 SD on the observed scale) and (b) between-vowel contrasts at each step. Family-wise error within each prespecified family was controlled with Holm adjustment.

**Reaction times.** RTs for pressed keys were analyzed on the log scale with linear mixed-effects models. Latencies were expressed in seconds, and trials with missing or extreme latencies were removed using 0.1–3.0 s bounds, followed by subject-wise ±3 median absolute deviation (MAD [45]) trimming on log-RT. Fixed-effect specification, factor coding (adjacent contrasts for Step; sum-coding for Vowel and Gender), and covariates (Age, Education, L2 Use, L2 Proficiency; all z-scored) mirrored the response analysis. Models were compared with ML LRTs while preserving hierarchy, and the final LMM was refit with REML, while fixed effects were evaluated with Satterthwaite degrees of freedom. Planned emmeans contrasts matched the response analysis (adjacent-step within vowel; between-vowel at each step). For interpretability, estimates and contrasts are reported as time ratios (exponentiated log-RT effects) with 95% CIs.

#### 4.3.2. AX Task: Sensitivity, Response Accuracy, and Reaction Times

We first quantify sensitivity and decision bias using signal-detection theory (SDT) tailored to the same–different AX design with roving stimuli (i.e., the SD differencing model) [46,47,48], and then we perform mixed-effects inference on discrimination accuracy and RTs. Many speech-perception papers summarize AX same–different task performance using yes/no-style *d*′ and *c* as a heuristic (e.g., [49,50]). However, we do not use those inferences here because the mapping from (*H*, *F*) to sensitivity/bias differs under roving SD.

**SDT indices (roving same–different, differencing model).** For each listener and condition (Vowel, Step distance Δ), we treat “different” vs. “same” responses as SDT outcomes with signals = different trials (A ≠ X) and noise = same trials (A = X). We use log-linear (Hautus) correction to avoid 0/1 rates by adding 0.5 to *H*, *FA*, *M*, *CR*, and 1 to the corresponding denominators [51]. Following the sensitivity and ROCs equations in [48], we let

$$H=\mathrm{Pr}\left(“\mathrm{different}”|\mathrm{different}\right),\;\;\;\;\;\;F=Pr(“\mathrm{different}”|\mathrm{same}).$$

Under the differencing rule appropriate for roving AX, bias is parameterized by a criterion on the difference axis, *k*, and sensitivity by *d*′:

$$H\;=\;\Phi \;\left(\frac{-k+d^{\prime }}{\sqrt{2}}\right)+\Phi \;\left(\frac{-k-d^{\prime }}{\sqrt{2}}\right),\;\;\;\;\;F\;=\;2\;\Phi \left(-\frac{k}{\sqrt{2}}\right)$$

with Φ the standard normal cumulative distribution function. We estimate

$$k\;=\;\sqrt{2}\;\Phi^{-1}\;(1-\frac{F}{2}),$$

then solve numerically for *d*′ from the *H* equation (monotone in *d*′, bounded root on [0, 10]) (using R script by [44]). As a complementary bias index, we report the log likelihood-ratio at the criterion for “different” vs. “same”,

$$ln\;\beta_{d}\;=\;-\frac{{d^{\prime }}^{2}}{4}+\;ln\;[cosh(\frac{k\;d^{\prime }}{2})],$$

which increases with a conservative “same” bias (larger *k*). For interpretability, we also report *k* directly (in SD units on the difference axis).

Since at Δ = 0, there are no signal trials, we do not compute *d*′ at Δ = 0 and report only *F* and the implied *k* (criterion). Additionally, we compute percentile-bootstrap 95% CIs across listeners (10,000 resamples) for *d*′ and *k*, and ln*β*_*d*_ within each Vowel × Δ.

**Response accuracy**. Trial-level accuracy (1 = correct, 0 = incorrect) was modeled with a binomial GLMM (lme4, logit link). Analyses were restricted to Δ = 2 “different” pairs; the pair midpoint (CV_1_–CV_5_) was coded as an ordered factor with successive-difference (adjacent) contrasts to allow non-linear discrimination across the continuum. Vowel (/i/, /a/) and the Vowel × Midpoint interaction were the primary design factors. Age and Education were entered as z-scored covariates; L2 Use, L2 Proficiency, and Gender were evaluated but not retained because they did not improve fit (model selection described below). Random effects comprised by-subject intercepts and a by-subject slope for Vowel. Models were compared by ML likelihood-ratio tests. Planned emmeans contrasts were (a) adjacent-Midpoint comparisons within each vowel and (b) between-vowel differences at each Midpoint, with Holm adjustment controlling family-wise error within each contrast family.

**Reaction times.** Analyses were restricted to Δ = 2 “different” trials and to correct responses. RTs < 0.1 s or >3 s were removed; remaining RTs were log-transformed and trimmed within participant at ±3 MAD around the participant’s median (on log-RT). A linear mixed-effects model predicted log-RT from Midpoint (CV_1_–CV_5_) × Vowel (/i/, /a/). Midpoint was treated as an ordered factor with successive-difference (adjacent) contrasts; Vowel was sum-coded. Age (z) and L2 use (z) were included as covariates (Education, L2 Proficiency, Gender were evaluated but not retained). Random effects comprised a by-subject intercept and by-subject Vowel slope; a by-token intercept was considered but omitted when it induced singular fits. Model selection used ML likelihood-ratio tests, the final model was refit with REML, and fixed effects were tested with Satterthwaite df. Planned emmeans contrasts were computed on the link (log) scale and reported as time ratios (TR = exp(β)) with Holm-adjusted inferences.

## 5. Results

### 5.1. Identification Task Responses

We analyzed categorical identification along the /n/–/l/ continua (CV_0_–CV_6_) in two vowel contexts (/i/, /a/). The dependent variable was the probability of choosing /l/ (1 = /l/, 0 = /n/). After preprocessing, 5326 of 5376 trials entered the analysis (32 listeners; 14 tokens); the 50 missing trials (0.93%) were trials on which participants did not provide a response within the response window (timeouts), so no valid response/RT was recorded. Trial balance was high: participants contributed on average 166.4 trials (SD = 1.8; range = 162–168), and each token accrued on average 380.4 trials (SD = 2.8; range = 375–384).

As detailed in Section 4.3.1, we fit binomial logistic mixed-effects models to trial-level /l/ responses, with Step, Vowel, and their interactions as primary predictors and z-scored Age, Education, L2 Use, and L2 Proficiency as covariates, plus by-subject random intercepts and Vowel slopes. Model selection proceeded via nested likelihood-ratio tests under maximum likelihood. Model comparison indicated reliable interactions of Vowel with Age (χ^2^(1) = 3.90, *p* = 0.048) and of Step with Age (χ^2^(6) = 47.42, *p* < 0.001). Gender terms (main effect, Vowel × Gender, Step × Gender) did not improve fit (all *p* > 0.05). Education improved the model both as a main effect (χ^2^(1) = 4.65, *p* = 0.031) and in interaction with Vowel (χ^2^(2) = 8.06, *p* = 0.018) and Step (χ^2^(7) = 26.31, *p* < 0.001). L2 Use (main effect, Vowel × Use, Step × Use) had no detectable contribution (all *p* > 0.05). L2 Proficiency did not shift overall means (main effect or Vowel × Proficiency, both *p* > 0.05) but did interact with Step (χ^2^(7) = 57.75, *p* < 0.001), indicating modulation of the identification function’s shape. The resulting model can be summarized as follows:

$$\begin{array}{l} \mathrm{Response}∼\mathrm{Step}\times \mathrm{Vowel}+\mathrm{Age}+\mathrm{Proficiency}+\mathrm{Education}+(\mathrm{Vowel}\times \mathrm{Age})+(\mathrm{Step}\times \\ \mathrm{Age})+(\mathrm{Step}\times \mathrm{Proficiency})+(\mathrm{Step}\times \mathrm{Education})+(1+\mathrm{Vowel}||\mathrm{Subject}). \end{array}$$

Diagnostics indicated a satisfactory fit: the model was not singular by standard checks; overdispersion was absent (dispersion ratio = 1.016, *p* = 0.816); and simulated DHARMa residuals showed no concerning structure. Model R^2^ was high for a categorical task (marginal R^2^ = 0.593, conditional R^2^ = 0.600). Random-effect variances by Subject were 0.051 for the intercept and 0.798/2.662 for Vowel slopes (see Supplementary Table S2.1 for fixed effects).

On the probability scale, the grand-mean intercept corresponded to P(/l/) = 0.678, indicating that /l/ responses were common overall. Averaged over steps, tokens in /i/ context elicited fewer /l/ responses than in /a/ context (OR = 0.387, z = −2.25, *p* = 0.025), i.e., the overall /l/ bias was stronger in /a/. At CV_3_ (midpoint), P(/l/) was 0.631 [0.537, 0.717] for /i/ and 0.731 [0.588, 0.838] for /a/, suggesting that the category boundary lies right of the midpoint for /i/ (more /n/ than /l/ at CV_3_) and is flatter and higher for /a/.

**Figure 2:**
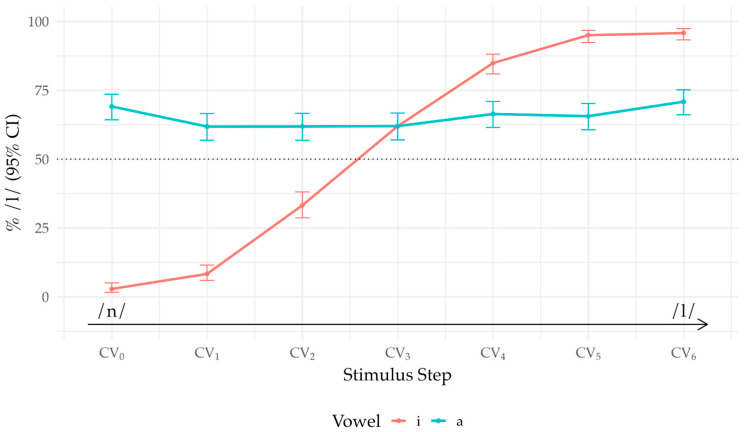
/n-l/ identification by vowel. The lines trace the percentage of /l/ responses across stimulus steps (CV_0_–CV_6_); bars are 95% Wilson CIs. Dotted line = 50%; arrow shows /n/ → /l/.

Fixed-effects estimates (Supplementary Table S2.4) showed robust Step × Vowel structure. In /i/, all adjacent-step increases except the last were significant (at mean covariates) after Holm adjustment: CV_0_ → CV_1_ (OR = 3.50, *p* = 0.002), CV_1_ → CV_2_ (OR = 7.20, *p* < 0.001), CV_2_ → CV_3_ (OR = 3.97, *p* < 0.001), CV_3_ → CV_4_ (OR = 4.22, *p* < 0.001), CV_4_ → CV_5_ (OR = 3.96, *p* < 0.001), whereas CV_5_ → CV_6_ was not (OR = 1.47, *p* = 0.304). In /a/, no adjacent contrast reached significance at the mean of covariates (all Holm-adjusted *p* > 0.05). This pattern suggests that the identification function is steep and asymmetric for /i/ (increasing as stimulus tokens shift from /n/ to /l/) but comparatively flat for /a/ (see Figure 2).

With respect to age, older listeners had higher grand-mean /l/ response rates (β = 0.412, z = 2.52, *p* = 0.012), with a Vowel × Age offset: older listeners produced relatively fewer /l/ responses in /i/ vs. /a/ (β = −0.475, z = −2.29, *p* = 0.022). Stepwise modulation by Age was limited; the most notable effect was a larger CV_0_ → CV_1_ change with age (β = 0.788, z = 3.79, *p* < 0.001), with other adjacent-step interactions non-significant.

Higher education reduced grand-mean /l/ responding (β = −0.390, z = −2.67, *p* = 0.008) and attenuated late-continuum changes: Step × Education was negative at 5 → 4 (β = −0.469, z = −2.77, *p* = 0.006) and 7 → 6 (β = −0.486, z = −2.13, *p* = 0.033), consistent with a boundary shift toward /n/ among more-educated listeners.

There was no reliable main effect of L2 proficiency, and stepwise tendencies were weak (largest at 5 → 4, *p* = 0.057), providing little evidence that L2 proficiency markedly altered adjacent-step slopes despite the significant global Step × Proficiency interaction. For transparency, fitted probabilities by Step × Vowel at covariate means and Holm-adjusted between-vowel tests at each step are provided in Supplementary Table S2.4. Within-vowel adjacent-step tests with Holm adjustment are provided in Supplementary Table S2.5. These tables report estimates on the probability and odds-ratio scales with back-transformed 95% CIs.

### 5.2. Identification Task RTs

We analyzed identification RTs from the same trials. Of 5326 trials contributing to the response analysis, 4931 remained for RT modeling (7.41% trimmed). Trials were excluded for missing RTs, values outside the 0.1–3.0 s bounds, and subject-wise outliers beyond ±3 MAD on log-RT.

As described in Section 4.3.1, log-RTs (in seconds) were modeled with linear mixed-effects models including Step and Vowel as fixed effects and z-scored individual-difference covariates. Through a sequence of ML likelihood-ratio tests, we selected a final specification of: logRT~Step × Vowel + Age + L2 Proficiency + (1 + Vowel | Subject). Adding Age improved fit relative to a Step × Vowel baseline (χ^2^(1) = 13.34, *p* < 0.001). Adding Gender improved fit over the baseline (χ^2^(1) = 6.13, *p* = 0.013) but not beyond the Age model (χ^2^(1) = 2.90, *p* = 0.089).

Education showed no detectable contribution as a main effect or in interaction (all *p* > 0.05). L2 Use showed a trend as a main effect (χ^2^(1) = 2.82, *p* = 0.093) with no interactions (all *p* > 0.05). L2 Proficiency improved fit as a main effect (χ^2^(1) = 12.67, *p* < 0.001), with no interactions (all *p* > 0.05). Age × Vowel and Age × Step were not supported (all *p* > 0.05). A combined Age + Proficiency model fit better than an Age-only model (χ^2^(1) = 4.65, *p* = 0.031). Fixed effects were evaluated with Satterthwaite degrees of freedom, and estimates are reported on the log scale with time ratios (TR = exp(β)) in Supplementary Table S3.1.

The grand-mean intercept corresponded to ~721 ms (exp(−0.328) s). Age was associated with slower responses (β = 0.086, t = 2.30, *p* = 0.029), a +8.96% increase in RT per +1 SD of age (TR = 1.090). L2 Proficiency predicted faster responses (β = −0.080, t = −2.13, *p* = 0.041), a −7.65% change per +1 SD (TR = 0.923). The Vowel main effect was small and not reliable (*p* > 0.05).

**Figure 3:**
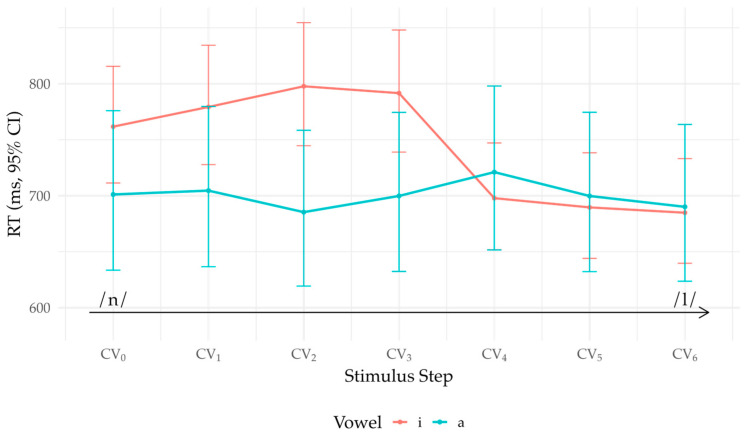
Identification response times (model-based means ± 95% CI) by continuum step and vowel. Estimates are back-transformed from the final mixed-effects model (log scale) and evaluated at Age = 0 and L2 Proficiency = 0 (z-scored means); arrow shows /n/ → /l/.

Stepwise effects were modest overall, with a localized change near the upper continuum. The only reliable adjacent-step main effect was 5 → 4 (β = −0.048, z = −2.79, *p* = 0.005), corresponding to a 4.7% speed-up averaged over vowels (TR = 0.953). The Step × Vowel interaction was similarly selective: the 5 → 4 × Vowel interaction was robust (β = −0.078, z = −4.52, *p* < 0.001), whereas other adjacent-step contrasts showed no reliable vowel modulation (all *p* > 0.05). Decomposition showed that the 5 → 4 transition was ~11.8% faster in /i/ (TR = 0.882) but ~3.0% slower in /a/ (TR = 1.030), indicating vowel-dependent behavior in the RT function near the /l/ end of the continuum. Between-vowel RT differences at individual steps were otherwise small and, after Holm adjustment, not systematically different from zero. Figure 3 visualizes model-based means (back-transformed to milliseconds with 95% CIs) by vowel across steps.

### 5.3. AX Discrimination (SDT, Differencing)

We estimated bias and sensitivity under the equal-variance differencing model appropriate for roving same–different AX (see Methods). For each Subject × Vowel, we anchored the criterion *k* from the false-alarm rate at Δ = 0 (“same pairs”, noise) and solved *d*′ at Δ = 2 (two-step “different” pairs) from the hit rate (signals).

**Figure 4:**
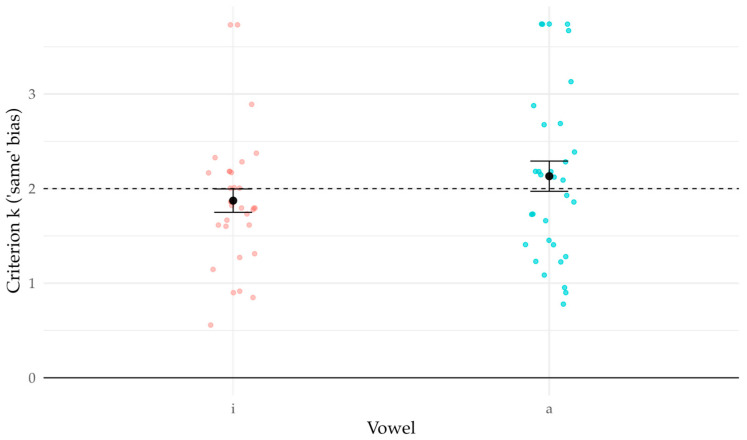
Response bias by vowel. Individual listeners (jittered points); black dot ± SE across listeners summarizes the between-listener variability in the group mean. Criterion *k* computed from Δ = 0 (“same”) trials; *k* = 0 indicates no bias (solid line), *k* = 2 shown for reference (dashed).

Pooled false-alarm rates were FA0 = 0.220 in /i/ and 0.196 in /a/. These imply positive criteria (consistent with a “same” bias) of *k*/*i*/ = 1.87 [1.64, 2.12] and *k*/*a*/ = 2.13 [1.83, 2.44] (Figure 4). Across Subject × Vowel cells, FA0 was generally well below 0.5 (median 0.189, IQR 0.107–0.307, max 0.693), indicating a robust “same” bias overall.

**Figure 5:**
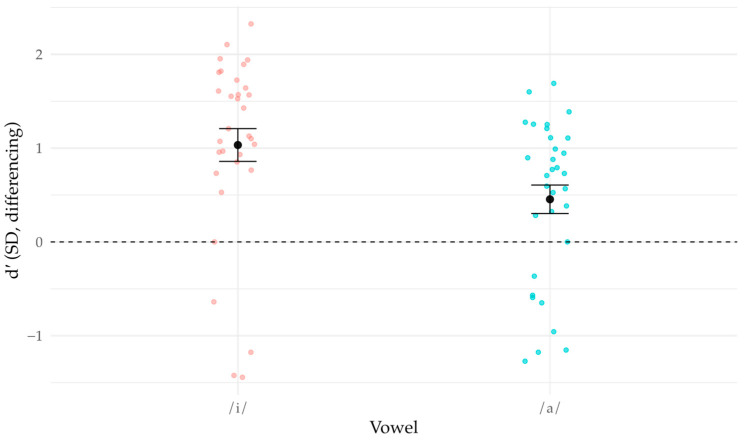
Sensitivity (d′) at Δ = 2. Individual listeners with mean ± SE by vowel (/i/, /a/). Higher d′ indicates better discriminability; d′ = 0 shown as a reference (dashed line).

Pooled hit rates were 0.365 in /i/ and 0.231 in /a/. Given the FA0 anchors above, differencing-model estimates were *d*′/*i*/ = 1.03 [0.68, 1.35] and *d*′/*a*/ = 0.45 [0.16, 0.74] (Figure 5). Negative *d*′ values, expected when *H* < FA0, occurred in 18.8% of Subject × Vowel cells.

**Figure 6:**
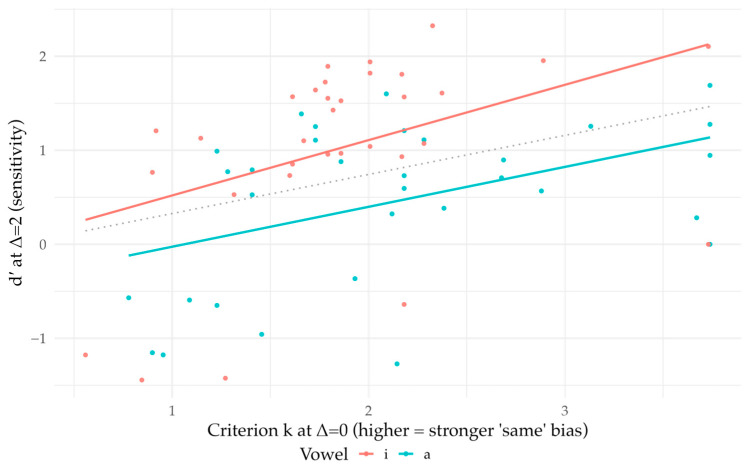
Bias–sensitivity relationship. Scatter of criterion *k* at Δ = 0 vs. d′ at Δ = 2 by vowel, with pooled OLS fit (gray, dotted) and per-vowel fits (colored). Each point is one listener.

Across listeners, criterion *k* and sensitivity *d*′ were positively associated (per-vowel OLS; see Figure 6), indicating that listeners who were more conservative (‘same’-biased; lower FA) also tended to be more sensitive at Δ = 2 (higher H). This between-subject pattern reflects joint variation in ability and response strategy but does not imply a within-subject trade-off.

### 5.4. AX Discrimination Accuracy

We analyzed AX discrimination at Δ = 2 (unordered 2-step pairs) in two vowel contexts (/i/, /a/), using trial-level accuracy as the dependent variable (1 = correct “different”, 0 = incorrect). After preprocessing and restricting to “different” trials only, 3748 trials entered the analysis.

As detailed in Section 4.3.2, we fit binomial logistic mixed-effects models with Midpoint (CV_1_–CV_5_), Vowel, and their interaction as primary predictors, z-scored Age and Education as covariates, and by-subject random intercepts with a Vowel slope. Model selection used nested likelihood-ratio tests (ML). Vowel × Age and the Age main effect did not improve fit (both *p* > 0.05). Nevertheless, Midpoint × Age improved fit (χ^2^(5) = 11.50, *p* < 0.05). Gender terms (main effect, Vowel × Gender, Midpoint × Gender) were uninformative (all *p* > 0.05). Education did not shift overall accuracy nor interact with Vowel, but Midpoint × Education improved fit (χ^2^(5) = 13.85, *p* = 0.016). Finally, L2 Use and L2 Proficiency (main effects and interactions with Vowel or Midpoint) showed no evidence of contribution (all *p* > 0.05). The resulting model was Accuracy∼Vowel × Midpoint + Age + Education + (Midpoint × Age) + (Midpoint × Education) + (1 + Vowel ∣∣ Subject).

Diagnostics indicated a satisfactory fit: no singularity by standard checks; no overdispersion (dispersion = 1.012, *p* = 0.872), and DHARMa residuals showed no concerning structure. Model R2 was marginal = 0.145, conditional = 0.241. Random-effect variances by Subject were 0.419 (intercept) and 0.271/0.896 for Vowel slopes.

On the probability scale, the grand-mean intercept corresponded to P(correct) = 0.241, reflecting overall task difficulty for “different” trials, when averaged across Midpoints and covariates. Averaged over Midpoints, listeners were more accurate in /i/ than in /a/ (OR = 2.29, z = 4.25, *p* < 0.001). Simple effects by Midpoint showed robust Vowel differences at CV_1_–CV_3_ (all Holm-adjusted *p* < 0.001; ORs ≈ 4.68, 5.70, 2.52), but no reliable difference at CV_4_–CV_5_ (both *p* > 0.05).

**Figure 7:**
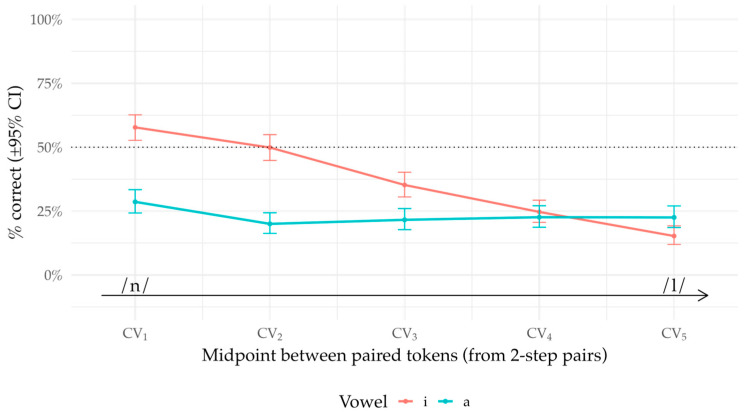
Discrimination accuracy at Δ = 2. The lines trace the percentage of correct for 2-step “different” pairs by midpoint (CV_1_–CV_5_) and vowel; error bars are 95% Wilson CIs. Dotted line marks 50% chance; arrow shows /n/ → /l/.

Fixed-effects estimates (Supplementary Table S4.1) revealed a clear Midpoint × Vowel structure. Within /i/, accuracy declined monotonically across the continuum: CV_2_ vs. CV_1_ (OR = 0.69, z = −2.32, *p* = 0.020), CV_3_ vs. CV_2_ (OR = 0.50, z = −4.30, *p* = 0.001), CV_4_ vs. CV_3_ (OR = 0.57, z = −3.25, *p* = 0.002), and CV_5_ vs. CV_4_ (OR = 0.52, z = −3.35, *p* = 0.002; Holm-adjusted). In /a/, only the early contrast (CV_2_ vs. CV_1_) reached significance (OR = 0.57, z = −3.03, *p* = 0.010), while later adjacent changes were not reliable (all Holm-adjusted *p* > 1.00). This pattern indicates a steep, graded loss of discriminability along the /i/ continuum but a comparatively flat function for /a/, as illustrated in Figure 7.

With respect to covariates, Age had no main effect on overall accuracy (β = −0.200, z = −1.30, *p* > 0.05) and did not interact with Vowel (*p* > 0.05), but did modulate late-continuum change: the CV_5_ → CV_4_ contrast grew more negative with Age (β = −0.301, z = −2.18, *p* = 0.029), suggesting older listeners show a sharper accuracy drop at the most /l/-like end. Education likewise showed no main effect (*p* > 0.05) and no Vowel interaction (*p* > 0.05) yet reliably interacted with Midpoint (global LRT *p* = 0.016). In /i/, the early drop (CV_2_ → CV_1_) weakens with Education: OR = 0.54 at −1 SD (*p* = 0.007), 0.69 at mean (*p* = 0.020), 0.89 at +1 SD (*p* = 0.57, n.s.). In /a/, stepwise changes are broadly flat beyond the first step (all Holm *p* > 0.64).

### 5.5. AX Discrimination RTs

We analyzed RTs from the Δ = 2 (“different”) AX trials. Of 3748 trials, 3323 remained after RT preprocessing (11.34% trimmed) and exclusions of missing RTs, values outside 0.1–3.0 s, and subject-wise outliers beyond ±3 MAD on log-RT. As in Section 4.3.1, log-RTs (s) were modeled using LMMs that included Midpoint (CV1–CV5) and Vowel as fixed effects, along with z-scored covariates. Model selection via ML LRTs favored adding Age and L2 Use to a Midpoint × Vowel baseline: Age improved fit (χ^2^(1) = 5.20, *p* = 0.023), and adding L2 Use to the Age model further improved fit (χ^2^(1) = 4.55, *p* = 0.033). Gender and Education did not contribute as main effects or interactions (all *p* > 0.05). L2 Proficiency improved fit over the baseline (χ^2^(1) = 5.26, *p* = 0.022) but did not improve an Age-only model (χ^2^(1) = 1.50, *p* > 0.05), so the final model was logRT~Midpoint × Vowel + Age + L2 Use + (1 + Vowel|Subject).

The grand-mean intercept corresponded to ≈ 489 ms (exp(−0.715) s). RTs were slower for older participants (β = 0.112, t = 2.19, *p* = 0.036), a +11.8% change per +1 SD of Age (TR = 1.12), and faster with greater L2 Use (β = −0.111, t = −2.18, *p* = 0.038), a −10.5% change per +1 SD (TR = 0.895). Vowel showed a small but reliable main effect (t = 3.24, *p* = 0.003), with /i/ responses slower than /a/ on average; however, neither Age × Vowel nor moderator × Midpoint interactions were supported (all *p* > 0.05).

**Figure 8:**
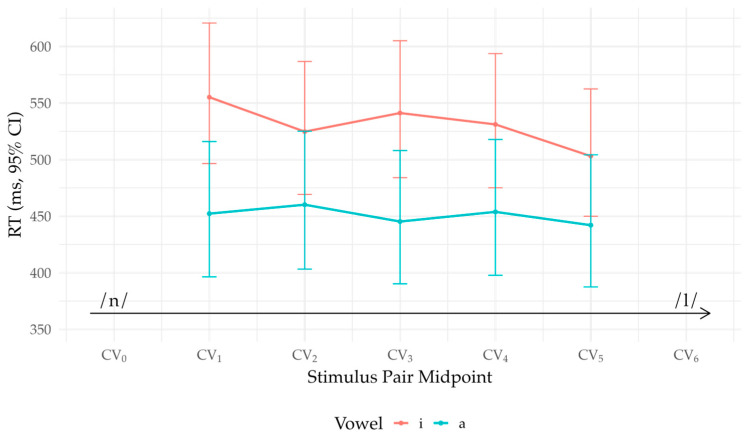
AX reaction times at Δ = 2. The lines trace model-based mean RTs (ms) by midpoint and vowel with 95% CIs; the arrow shows /n/ → /l/.

Stepwise effects across midpoints were modest: no adjacent Midpoint contrast reached Holm-adjusted significance (all *p* > 0.05), and the Midpoint × Vowel interaction was not reliable (all *p* > 0.05). Consistent with this, between-vowel differences at individual midpoints were small and did not survive Holm correction. Figure 8 plots model-based means (back-transformed to milliseconds with 95% CIs) by vowel across midpoints.

## 6. Discussion

The present study examined how Sichuan Mandarin listeners perceive the /n-l/ contrast using spectrally morphed continua in two vowel contexts, assessed with categorical identification and same–different AX discrimination. Across tasks, listeners showed overall reduced sensitivity to the contrast, consistent with overlap between /n/ and /l/ reported for Southwestern Mandarin varieties. At the same time, perceptual performance was not uniform across contexts: listeners consistently showed stronger differentiation in the /i/ continuum than in the /a/ continuum. Individual differences related to age, education, and Standard Mandarin experience modulated response efficiency and bias, but did not eliminate the robust vowel-dependent asymmetry observed at the group level.

The perceptual advantage observed in the /i/ context is consistent with the acoustic structure of the stimuli. In the /i/ continuum, amplitude-based measures exhibited larger magnitudes and clearer grading across continuum steps, providing listeners with more informative and separable acoustic differences between /n/ and /l/. In contrast, these measures spanned a compressed range in the /a/ continuum, and first-formant bandwidth did not vary systematically across steps in either vowel context. Prior work has shown that perceptual sensitivity to nasal–lateral contrasts depends on the availability and reliability of acoustic cues rather than on any single invariant correlate [2,29,30]. From this perspective, reduced cue separability in the /a/ context plausibly limited perceptual differentiation, whereas the more pronounced amplitude-based differences in /i/ supported greater contrast sensitivity. More broadly, this pattern is consistent with models of speech perception in which listeners dynamically weight multiple, non-invariant acoustic cues based on their relative reliability within a given phonetic context, rather than relying on any single stable correlate [52,53,54].

Crucially, however, the presence of measurable acoustic differences does not guarantee perceptual distinctiveness. A substantial body of work on nasality perception demonstrates that perceived nasalization depends on the integration of multiple cues—particularly in the F1 region—and that vowel context can strongly modulate how these cues are weighted and interpreted (e.g., [19,55,56]). From this perspective, the weak discrimination observed in the /a/ continuum likely reflects the dispersion or impoverishment of contrast-relevant cues in the spectrally morphed stimuli, rather than a categorical absence of perceptual sensitivity. The fact that listeners showed merger-like behavior in /a/ despite longer vowels and residual acoustic structure underscores the importance of cue coherence, not just cue magnitude, for successful perceptual differentiation.

From a signal-detection perspective, this pattern constrains how the /n-l/ contrast is represented in the perceptual system. In roving same–different AX tasks, performance can be limited either by decision criteria or by the separability of the internal sensory representations evoked by the stimuli. The present SDT results show that although listeners adopted a conservative “same” bias in both vowel contexts, this bias did not differ systematically between /i/ and /a/, whereas sensitivity (d′) was markedly reduced in /a/. This dissociation indicates that the vowel asymmetry does not arise from changes in decision strategy but from differences in the underlying perceptual evidence available for discriminating /n/ and /l/. Had the asymmetry reflected response strategy or task difficulty, corresponding changes in response bias would be expected across contexts; the absence of such changes therefore constrains the effect to the level of perceptual encoding. In SDT terms, the internal response distributions corresponding to /n/ and /l/ overlap much more strongly in the /a/ context than in the /i/ context, yielding merger-like behavior even though some acoustic differences remain in the signal. Crucially, this implies that near-merger in Sichuan Mandarin reflects compression of contrastive information at the level of perceptual encoding rather than categorical loss of phonological knowledge: when the acoustic input provides coherent cue structure, as in the /i/ continuum, listeners can recover partial separation between the categories, but when cues are weak or diffusely distributed, the internal representations collapse into a highly overlapping perceptual space.

The direction of the vowel asymmetry observed here should therefore not be interpreted as a general property of /n-l/ perception. Using speeded AX discrimination with Standard Mandarin listeners, Liu and Li [18] found that the /n-l/ contrast was perceptually more distinct in the /a/ context than in the /i/ context when both were produced with an identical high-level tone, a pattern opposite to that observed in the present study. Rather than indicating a contradiction, this difference highlights the context-dependent nature of vowel effects in speech perception: differences in stimulus construction, task demands, and listener phonological background can substantially influence which acoustic cues are most informative and how they are weighted. In the present study, although the /i/ continuum (/ni–li/) and the /a/ continuum (/na–la/) differed in lexical tone, tone was held constant across steps within each continuum and therefore cannot account for graded perceptual patterns or category structure within contexts. Moreover, prior work indicates that tone does not show robust effects on the realization of /n/ and /l/ in production in Southwestern Mandarin [21], and vowel-dependent perceptual asymmetries have been reported even when tone is experimentally controlled [18]. Taken together, these considerations suggest that the vowel-dependent asymmetry observed here is best understood as reflecting differences in segmental cue structure across the two spectrally morphed continua, rather than tone-driven or vowel-intrinsic perceptual advantages.

Signal detection analyses further revealed substantial individual variability. Listeners exhibited a strong same-response bias in both vowel contexts, and the positive association between sensitivity and bias across listeners suggests stable individual differences in perceptual clarity rather than a simple speed–accuracy trade-off. These differences likely reflect variation in how reliably listeners extract and integrate contrast-relevant cues from the acoustic signal, without altering the overall vowel-dependent pattern observed at the group level.

Individual differences related to age, education, and Standard Mandarin experience shaped perceptual performance without redefining the overall context-dependent representational pattern. Age was associated with slower responses and increased /l/-biased responding, consistent with age-related changes in the encoding or weighting of fine-grained spectral cues. Education exerted a stabilizing effect on category use and response efficiency, plausibly reflecting greater exposure to Standard Mandarin norms and more consistent mapping between acoustic cues and phonological categories. Standard Mandarin proficiency and use showed more limited effects, primarily influencing response speed rather than discrimination accuracy or identification slopes. Crucially, none of these factors eliminated the vowel-dependent asymmetry in sensitivity, indicating that sociolinguistic experience modulates how listeners exploit available perceptual evidence rather than restoring a context-independent separation between /n/ and /l/.

This pattern aligns with findings from other /n-l/ merger systems. In Fuzhou Min, Cheng et al. [2] report near-complete perceptual merger despite residual acoustic differentiation in production, with younger listeners showing modest gains in sensitivity attributable to increased Mandarin exposure. The present results converge with this account while highlighting the role of cue availability: Sichuan Mandarin listeners showed measurable sensitivity when the acoustic signal instantiated sufficiently robust and coherent cues, but exhibited merger-like behavior when cues were weaker or more diffusely distributed. In this respect, the present study extends prior merger work by showing that perceptual differentiation can emerge selectively within a near-merger when the acoustic signal provides sufficiently coherent cue structure, even in the absence of robust category-level separation. Mandarin experience thus appears to facilitate selective cue exploitation rather than categorical recovery of the contrast.

Taken together, the results support an account of the /n-l/ alternation in Sichuan Mandarin as a context-dependent near-merger, in which perceptual differentiation is reduced overall but can emerge under favorable acoustic conditions. Perception is shaped jointly by the structure of the acoustic signal, phonetic context, and listeners’ experience with a contrastive system. These findings underscore the importance of integrating detailed acoustic analysis with perceptual data in studies of merger and caution against interpreting context-dependent discrimination failures as direct evidence of categorical phonological neutralization.

We note one limitation of the present design. Although the talker was a highly proficient speaker of Standard Mandarin and did not exhibit an /n-l/ merger in the selected materials, she was not a native speaker of Standard Mandarin. As a result, it is possible that the acoustic realization of the /n-l/ contrast—particularly in the /a/ context—may not fully reflect the canonical production norms of native Standard Mandarin, potentially limiting the extent to which the present continua capture the full cue structure available in that vowel context.

## 7. Conclusions and Future Directions

The present study shows that Sichuan Mandarin listeners’ perception of the /n-l/ contrast is strongly context-dependent, with substantially greater sensitivity in a spectrally morphed /i/ continuum than in a corresponding /a/ continuum. Although age, education, and Standard Mandarin experience modulated response efficiency and bias, these factors did not reverse the overall vowel asymmetry. Acoustic analyses indicate that the /i/ continuum instantiated more robust and coherent segmental cue differences than the /a/ continuum, suggesting that perceptual outcomes reflect listeners’ ability to exploit available acoustic structure rather than uniform preservation of the contrast across contexts. Together, the findings support a view of /n-l/ merger in Sichuan Mandarin as gradient and context-dependent, shaped by cue availability and listener experience rather than categorical loss or recovery of a phonological contrast. In addition, future work should replicate the present paradigm using (a) native Standard Mandarin talkers and (b) Sichuan Mandarin talkers to directly assess how talker- and dialect-specific cue encoding modulates perceptual recoverability, particularly in contexts such as /a/ where cue structure may be less robust.

An important next step is to examine whether the perceptual asymmetry observed here is mirrored in speakers’ productions. Ongoing analyses of the corresponding production data will assess the extent to which /n-l/ realizations differ across vowel contexts and whether residual acoustic differentiation aligns with the perceptual patterns reported here. Directly linking production and perception within the same speaker population will help clarify whether listeners’ sensitivity reflects properties of their own speech or selective exploitation of cues present in the acoustic signal. More broadly, extending this approach to additional vowel contexts, tonal environments, and task paradigms will further illuminate how cue structure and phonetic context jointly shape perception in near-merger systems. Such work will contribute to a more nuanced understanding of how gradient phonetic variation, rather than categorical neutralization, underlies patterns of sound change and stability in spoken language.

## Figures and Tables

**Table 1:** Consonant and vowel durations, ΔA1, relative RMS amplitude, and mean BW1 values across the seven-step /n-l/ continua (from CV_0_ to CV_6_, where CV_0_ = /n/ endpoint and CV_6_ = /l/ endpoint) in the /i/ and /a/ vowel contexts.

	Step	C Duration (ms)	V Duration (ms)	Relative RMS Amplitude (dB)	Δ A1 (dB)	BW1 (Hz)
/ni/-/li/	CV_0_	77.162	322.807	17.485	47.531	135.61
	CV_1_	76.629	322.804	14.827	40.352	118.603
	CV_2_	77.590	322.432	14.111	37.793	134.511
	CV_3_	76.993	323.030	12.741	36.694	134.452
	CV_4_	77.321	322.706	12.493	35.279	160.843
	CV_5_	76.586	326.022	11.591	34.805	162.621
	CV_6_	74.845	321.821	11.007	34.424	134.845
/na/-/la/	CV_0_	71.162	428.069	7.482	2.35	122.772
	CV_1_	71.261	428.092	7.769	2.706	125.66
	CV_2_	71.133	428.092	8.061	3.141	125.574
	CV_3_	71.195	428.092	8.376	3.659	125.315
	CV_4_	71.289	428.092	8.707	4.286	126.269
	CV_5_	71.263	428.092	9.075	5.023	126.099
	CV_6_	71.600	428.092	9.52	5.982	125.869

## Data Availability

The audio stimuli and the Python scripts used to generate them are available via the Open Science Framework (OSF) project at OSF|Perception of /n-l/ in Sichuan Mandarin. De-identified behavioral data and analysis scripts are available from the corresponding author upon reasonable request due to participant privacy and ethical restrictions.

## Supplementary Materials

The following supporting information can be downloaded at: https://www.mdpi.com/article/10.3390/brainsci16020155/s1, Supplementary Materials S1: Experimental Instructions (as displayed on screen prior to the experiment in Standard Mandarin (with English translations), including S1.1 instructions for the AX same–different discrimination task and S1.2 instructions for the forced-choice identification task; Table S2.1: Fixed effects from the GLMM analysis of identification responses (logit estimates and odds ratios with 95% CIs); Table S2.2: Overall vowel difference (averaged over steps); Table S2.3: Midpoint estimates (Step = 3); Table S2.4: Fitted P(/l/) by Step × Vowel (with Holm-adjusted vowel *p*-values per step); Table S2.5: Adjacent-step contrasts within vowel (Holm-adjusted); Table S3.1: Fixed effects on log-RT and time ratios (95% CI) from the LMM analysis of identification RT; Table S3.2: Between-vowel comparisons at each step for RT (log scale; Holm-adjusted); Table S3.3: Adjacent-step RT contrasts by Age level; Table S4.1: Fixed effects from the GLMM analysis of AX discrimination accuracy (logit estimates and odds ratios with 95% CIs); Table S4.2: Mean “different” accuracy by vowel (probability scale); Table S4.3: Estimated means by Midpoint × Vowel (probability scale; covariates at mean); Table S4.4: Adjacent midpoint contrasts within vowel (Holm-adjusted); Table S4.5: Vowel differences at each midpoint (Holm-adjusted); Table S5.1: Fixed effects on log-RT and time ratios (95% CI) from the LMM analysis of AX RT; Table S5.2: Between-vowel RT differences at each midpoint (Holm-adjusted); Table S5.3: Adjacent-midpoint RT changes by moderator level (Age; L2 Use).

## References

[1] Maguire W., Clark L., Watson K. (2013). Introduction: What Are Mergers and Can They Be Reversed?. Engl. Lang. Linguist..

[2] Cheng R., Jongman A., Sereno J.A. (2023). Production and Perception Evidence of a Merger: *l* and *n* in Fuzhou Min. Lang. Speech.

[3] Soo R., Johnson K.A., Babel M. Sound change in spontaneous bilingual speech: A corpus study on the Cantonese n-l merger in Cantonese-English bilinguals. Proceedings of the Interspeech 2021.

[4] Johnson K., Song Y. (2016). Gradient Phonemic Contrast in Nanjing Mandarin. J. Acoust. Soc. Am..

[5] Shi X., Xiang N. (2010). An Investigation of Sound Nasality in the Wuhan Dialect. Stud. Lang. Linguist..

[6] Zhang W., Levis J.M. (2021). The Southwestern Mandarin /n/–/l/ Merger: Effects on Production in Standard Mandarin and English. Front. Commun..

[7] Li L. (2009). On the Classification of Southwestern Mandarin. Fangyan.

[8] Dialect Investigation Group of Sichuan University (1960). The Phonological System of the Sichuan Dialect. J. Sichuan Univ..

[9] Ma C.D., Tan L.H. (1998). Comparison Study of Sichuan Dialect Phonetics and English Phonetics. J. Sichuan Teach. Coll..

[10] Zhang W. (2007). Alternation of [n] and [l] in Sichuan Dialect, Standard Mandarin and English: A Single-Case Study. Leeds Work. Pap. Linguist. Phon..

[11] Shi X. (2015). The Nasality of Sonorants in Chengdu Dialect: On the Nature and Type of the /n/–/l/ Merger. Chin. J. Phon..

[12] Wang W.H. (1994). The Phonetic Features of Putonghua with Sichuan Accent. J. Sichuan Univ..

[13] Shi X., Ran Q., Shi F. (2010). A Preliminary Analysis of Nasalance in Beijing Mandarin. Contemp. Linguist..

[14] Ng C.L.C. (2017). Merger of the Syllable-Initial [n-] and [l-] in Hong Kong Cantonese. Outstanding Academic Papers by Students.

[15] Yeung S.W. (1980). Some Aspects of Phonological Variations in the Cantonese Spoken in Hong Kong. Ph.D. Dissertation.

[16] Tong K.S.T., James G. (1994). Colloquial Cantonese.

[17] Chan A.Y.W. (2011). The Perception of English Speech Sounds by Cantonese ESL Learners in Hong Kong. TESOL Q..

[18] Liu P.B., Li M. (2024). The Perceptual Distinctiveness of the [n-l] Contrast in Different Vowel and Tonal Contexts. JASA Express Lett..

[19] Hawkins S., Stevens K.N. (1985). Acoustic and Perceptual Correlates of the Non-Nasal–Nasal Distinction for Vowels. J. Acoust. Soc. Am..

[20] Ziegler J.C., Ferrand L. (1998). Orthography Shapes the Perception of Speech: The Consistency Effect in Auditory Word Recognition. Psychon. Bull. Rev..

[21] Perfetti C.A., Liu Y. (2005). Orthography to Phonology and Meaning: Comparisons across and within Writing Systems. Read. Writ..

[22] Marian V., Blumenfeld H.K., Kaushanskaya M. (2007). The Language Experience and Proficiency Questionnaire (LEAP-Q): Assessing Language Profiles in Bilinguals and Multilinguals. J. Speech Lang. Hear. Res..

[23] Boersma P., Weenink D. (2025). Praat: Doing Phonetics by Computer, Version 6.4.51; Computer Program. https://www.praat.org.

[24] van Hessen A. Generation of natural sounding speech stimuli by means of linear cepstral interpolation. Proceedings of the 2nd International Conference on Spoken Language Processing (ICSLP 1992).

[25] Gerrits P.A.M. (2001). The Categorisation of Speech Sounds by Adults and Children: De Classificatie van Spraakklanken Door Volwassen Luisterars en Kinderen.

[26] Gerrits E., Schouten M.E.H. (2004). Categorical perception depends on the discrimination task. Percept. Psychophys..

[27] Allen J.B., Rabiner L.R. (1977). A unified approach to short-time Fourier analysis and synthesis. Proc. IEEE.

[28] Griffin D., Lim J. (1984). Signal estimation from modified short-time Fourier transform. IEEE Trans. Acoust. Speech Signal Process..

[29] Chen M.Y. (1997). Acoustic Correlates of English and French Nasalized Vowels. J. Acoust. Soc. Am..

[30] Styler W. (2017). On the Acoustical Features of Vowel Nasality in English and French. J. Acoust. Soc. Am..

[31] Peirce J.W., Gray J.R., Simpson S., MacAskill M.R., Höchenberger R., Sogo H., Kastman E., Lindeløv J. (2019). PsychoPy2: Experiments in Behavior Made Easy. Behav. Res. Methods.

[32] Werker J.F., Logan J.S. (1985). Cross-Language Evidence for Three Factors in Speech Perception. Percept. Psychophys..

[33] R Development Core Team (2023). R: A Language and Environment for Statistical Computing.

[34] Wickham H., Averick M., Bryan J., Chang W., McGowan L.-D., François R., Grolemund G., Hayes A., Henry L., Hester J. (2019). Welcome to the Tidyverse. J. Open Source Softw..

[35] Wickham H. (2016). Ggplot2: Elegant Graphics for Data Analysis.

[36] Bates D., Mächler M., Bolker B., Walker S. (2015). Fitting Linear Mixed-Effects Models Using lme4. J. Stat. Softw..

[37] Kuznetsova A., Brockhoff P.B., Christensen R.H.B. (2017). lmerTest Package: Tests in Linear Mixed Effects Models. J. Stat. Softw..

[38] Lenth R. (2023). Emmeans: Estimated Marginal Means, aka Least-Squares Means; R Package Version 1.8.4–1. https://CRAN.R-project.org/package=emmeans.

[39] Holm S. (1979). A Simple Sequentially Rejective Multiple Test Procedure. Scand. J. Stat..

[40] Hartig F. (2024). DHARMa: Residual Diagnostics for Hierarchical Regression Models; R Package Version 0.4.7. https://CRAN.R-project.org/package=DHARMa.

[41] Lüdecke D., Ben-Shachar M., Patil I., Waggoner P., Makowski D. (2021). performance: An R Package for Assessment, Comparison and Testing of Statistical Models. J. Open Source Softw..

[42] Venables W.N., Ripley B.D. (2002). Modern Applied Statistics with S.

[43] Powell M.J.D. (2009). The BOBYQA Algorithm for Bound Constrained Optimization Without Derivatives.

[44] Pallier C. (2002). Computing Discriminability (A′, d′) and Bias with R. Online Technical Note. https://www.pallier.org/pdfs/aprime.pdf.

[45] Leys C., Ley C., Klein O., Bernard P., Licata L. (2013). Detecting Outliers: Do Not Use Standard Deviation around the Mean, Use Absolute Deviation around the Median. J. Exp. Soc. Psychol..

[46] Sorkin R.D. (1962). Extension of the Theory of Signal Detectability to Matching Procedures in Psychoacoustics. J. Acoust. Soc. Am..

[47] Macmillan N.A., Creelman C.D. (1991). Detection Theory: A User’s Guide.

[48] Hautus M.J. (2022). Detection Theory: A User’s Guide.

[49] Babel M., McAuliffe M., Norton C., Senior B., Vaughn C. (2019). The Goldilocks Zone of Perceptual Learning. Phonetica.

[50] Zhao J., Yan H., Chien Y.F. (2025). The Production and Perception of Low Tone Alternations in Huaiyuan Chinese. Lab. Phonol..

[51] Hautus M.J. (1995). Corrections for Extreme Proportions and Their Biasing Effects on Estimated Values of *d*′. Behav. Res. Methods Instrum. Comput..

[52] Holt L.L., Lotto A.J. (2006). Cue Weighting in Auditory Categorization: Implications for First and Second Language Acquisition. J. Acoust. Soc. Am..

[53] Toscano J.C., McMurray B. (2010). Cue Integration with Categories: Weighting Acoustic Cues in Speech Using Unsupervised Learning and Distributional Statistics. Cogn. Sci..

[54] McMurray B., Jongman A. (2011). What Information Is Necessary for Speech Categorization? Harnessing Variability in the Speech Signal by Integrating Cues Computed Relative to Expectations. Psychol. Rev..

[55] Krakow R.A., Beddor P.S., Goldstein L.M., Fowler C.A. (1988). Coarticulatory Influences on the Perceived Height of Nasal Vowels. J. Acoust. Soc. Am..

[56] Beddor P.S., Krakow R.A. (1999). Perception of Coarticulatory Nasalization by Speakers of English and Thai: Evidence for Partial Compensation. J. Acoust. Soc. Am..

