# Supplementary material for: Near-Merger and Contextual Sensitivity in the Perception of /n-l/ in Sichuan Mandarin
Source: Brain Sci. 2026 Jan 29;16(2):155. doi: 10.3390/brainsci16020155 (PMC12938059; doi:10.3390/brainsci16020155)
Supplement: Supplementary file 1 [file brainsci-16-00155-s001.zip › brainsci-4121828-supplementary.pdf]

Supplementary Materials

S1. Experimental Instructions (as displayed on screen prior to the experiment)

The experimental instructions were presented to participants in Standard Mandarin. The Mandarin text below reproduces the instructions exactly as they appeared on screen during the experiment. English translations are provided for reference only and were not shown to participants.

S1.1 Instructions for the AX discrimination task

> 您将听到两个声音，请判断它们是否相同。
> 请尽量注意两个声音之间最细微的差别，
> 有时差别会比较明显，有时可能不太容易察觉。
> 按 ’BLUE’ 键： =
> 按 ’RED’ 键： ≠
> 每对声音只能按一次键。
> 如果没有按键，程序会自动进入下一题，
> 请不要尝试“修正”之前的答案。
> 请尽快做出反应，并保持专注。
> 请按空格键开始。
>
> ***English translation***
> You will hear two sounds. Please decide whether they are the same or different.
> Try to pay attention to even the smallest differences between the two sounds.
> Sometimes the difference will be obvious; sometimes it may be difficult to perceive.
> Press the 'BLUE' key: same (=)
> Press the 'RED' key: different (≠)
> You may press a key only once for each pair of sounds.
> If no key is pressed, the program will automatically proceed to the next trial.
> Please do not attempt to change a previous response.
> Please respond as quickly as possible and remain focused.
> Press the space bar to begin.

S1.2 Instructions for the forced-choice identification task

> 您将听到一个声音，请根据它属于哪一类选择对应的图像。
> 屏幕左侧和右侧将显示两张图片，它们的位置会随机变化。

按’BLUE’键选择左侧图像
按’RED’键选择右侧图像
每次听到声音后，请尽快按下您选择的按键。
请保持专注，并且每个声音只能按一次键。
如果您没有按键，将自动进入下一题，
请不要尝试“修正”之前的答案。
请按空格键开始。

English translation

You will hear a sound. Please choose the image corresponding to the category to which the sound belongs.
Two images will appear on the left and right sides of the screen; their positions will vary randomly.
Press the 'BLUE' key to select the image on the left.
Press the 'RED' key to select the image on the right.
After hearing each sound, please press your chosen key as quickly as possible.
Please remain focused, and note that only one key press is allowed for each sound.
If no key is pressed, the program will automatically proceed to the next trial.
Please do not attempt to change a previous response.
Press the space bar to begin.

S2. Summary of GLMM Analysis of Identification Responses

Table S2.1 Fixed effects (logit estimates and odds ratios with 95% CIs)

Term	Estimate (logit)	SE	OR	OR (low)	OR (high)	p
(Intercept)	0.745	0.138	2.105	1.606	2.761	< .001
Step (2–1)	0.425	0.215	1.530	1.004	2.330	0.048
Step (3–2)	1.002	0.151	2.722	2.026	3.658	< .001
Step (4–3)	0.713	0.123	2.041	1.604	2.596	< .001
Step (5–4)	0.883	0.134	2.418	1.861	3.142	< .001
Step (6–5)	0.681	0.175	1.976	1.402	2.786	< .001
Step (7–6)	0.385	0.213	1.470	0.967	2.232	0.071
Vowel (/i/ vs. /a/)	−0.475	0.211	0.622	0.411	0.941	0.025
Age	0.412	0.164	1.509	1.095	2.079	0.012
Proficiency	0.066	0.173	1.068	0.761	1.499	0.703
Education	−0.390	0.146	0.677	0.508	0.901	0.008
Step (2–1) × Vowel	0.827	0.211	2.286	1.512	3.455	< .001
Step (3–2) × Vowel	0.972	0.147	2.643	1.980	3.527	< .001
Step (4–3) × Vowel	0.666	0.121	1.947	1.536	2.469	< .001
Step (5–4) × Vowel	0.557	0.131	1.745	1.349	2.257	< .001
Step (6–5) × Vowel	0.695	0.172	2.004	1.430	2.808	< .001
Step (7–6) × Vowel	−0.002	0.207	0.998	0.666	1.496	0.993
Vowel × Age	−0.475	0.208	0.622	0.414	0.935	0.022
Step (2–1) × Age	0.788	0.208	2.199	1.462	3.306	< .001
Step (3–2) × Age	−0.083	0.183	0.921	0.644	1.317	0.651
Step (4–3) × Age	0.289	0.162	1.335	0.971	1.835	0.075
Step (5–4) × Age	0.021	0.178	1.021	0.720	1.448	0.908
Step (6–5) × Age	0.178	0.207	1.194	0.796	1.793	0.392
Step (7–6) × Age	−0.220	0.224	0.803	0.518	1.244	0.325
Step (2–1) × Proficiency	0.012	0.261	1.012	0.606	1.689	0.964
Step (3–2) × Proficiency	0.384	0.226	1.468	0.942	2.288	0.090
Step (4–3) × Proficiency	0.245	0.182	1.277	0.894	1.825	0.179
Step (5–4) × Proficiency	0.373	0.196	1.452	0.989	2.132	0.057
Step (6–5) × Proficiency	−0.016	0.222	0.985	0.637	1.521	0.944
Step (7–6) × Proficiency	0.446	0.251	1.562	0.955	2.555	0.076
Step (2–1) × Education	0.313	0.213	1.367	0.900	2.078	0.143
Step (3–2) × Education	−0.182	0.180	0.833	0.585	1.186	0.311
Step (4–3) × Education	−0.136	0.154	0.873	0.646	1.180	0.377

Step (5–4) × Education	−0.469	0.170	0.625	0.449	0.872	0.006
Step (6–5) × Education	−0.025	0.200	0.975	0.659	1.443	0.900
Step (7–6) × Education	−0.486	0.228	0.615	0.393	0.962	0.033

Table S2.2 Overall vowel difference (averaged over steps)

Contrast	Odds ratio (i / a)	SE	z	p
i / a	0.387	0.163	-2.249	0.025

Table S2.3 Midpoint estimates (Step = 3)

Step	Vowel	P(/l/)	SE	95% CI
3	i	0.631	0.0465	[0.537, 0.717]
3	a	0.731	0.0645	[0.588, 0.838]

Table S2.4 Fitted P(/l/) by step × vowel (with Holm-adjusted vowel p-values per step)

Step	Vowel	Prob	SE	LCL	UCL	p (/i/ vs. /a/)
0	i	0.017	0.006	0.008	0.034	< .001
0	a	0.790	0.055	0.664	0.877	< .001
1	i	0.057	0.014	0.035	0.091	< .001
1	a	0.716	0.067	0.569	0.827	< .001
2	i	0.301	0.042	0.225	0.390	< .001
2	a	0.722	0.066	0.577	0.831	< .001
3	i	0.632	0.046	0.537	0.717	0.301
3	a	0.731	0.065	0.588	0.838	0.301
4	i	0.879	0.024	0.823	0.919	0.154
4	a	0.790	0.055	0.664	0.878	0.154
5	i	0.966	0.010	0.940	0.981	< .001
5	a	0.788	0.055	0.660	0.877	< .001
6	i	0.977	0.007	0.957	0.988	< .001
6	a	0.845	0.044	0.739	0.913	< .001

Table S2.5 Adjacent-step contrasts within vowel (Holm-adjusted)

Vowel	From step	To step	OR	SE	z	p
i	CV_0	CV_1	3.496	1.338	3.270	0.002
i	CV_1	CV_2	7.196	1.693	8.386	< .001
i	CV_2	CV_3	3.974	0.656	8.354	< .001
i	CV_3	CV_4	4.219	0.818	7.422	< .001
i	CV_4	CV_5	3.960	1.168	4.666	< .001
i	CV_5	CV_6	1.467	0.546	1.028	0.304

a	CV_0	CV_1	0.669	0.125	−2.158	0.185
a	CV_1	CV_2	1.030	0.188	0.162	1.000
a	CV_2	CV_3	1.048	0.188	0.261	1.000
a	CV_3	CV_4	1.386	0.250	1.807	0.283
a	CV_4	CV_5	0.986	0.181	−0.076	1.000
a	CV_5	CV_6	1.472	0.286	1.994	0.231

S3. Summary of LMM Analysis of Identification RT

Table S3.1 Fixed effects on log-RT and time ratios (95% CI)

Term	Estimate (log)	SE	Time Ratio	95% CI	t / z	p
(Intercept)	−0.328	0.036	0.721	[0.669, 0.776]	−9.022	<.001
Step (2–1)	0.014	0.017	1.014	[0.980, 1.049]	0.806	0.420
Step (3–2)	−0.002	0.017	0.998	[0.965, 1.032]	−0.123	0.902
Step (4–3)	0.007	0.017	1.007	[0.973, 1.042]	0.380	0.704
Step (5–4)	−0.048	0.017	0.953	[0.921, 0.986]	−2.787	0.005
Step (6–5)	−0.021	0.017	0.979	[0.947, 1.013]	−1.219	0.223
Step (7–6)	−0.010	0.017	0.990	[0.957, 1.023]	−0.608	0.543
Vowel (/i/ vs. /a/)	0.029	0.019	1.029	[0.989, 1.071]	1.489	0.147
Age	0.086	0.037	1.090	[1.010, 1.176]	2.299	0.029
Proficiency	−0.080	0.037	0.923	[0.856, 0.997]	−2.133	0.041
Step (2–1) × Vowel	0.009	0.017	1.009	[0.976, 1.044]	0.521	0.602
Step (3–2) × Vowel	0.026	0.017	1.026	[0.992, 1.061]	1.479	0.139
Step (4–3) × Vowel	−0.014	0.017	0.986	[0.953, 1.020]	−0.822	0.411
Step (5–4) × Vowel	−0.078	0.017	0.925	[0.894, 0.957]	−4.523	<.001
Step (6–5) × Vowel	0.009	0.017	1.009	[0.976, 1.044]	0.528	0.598
Step (7–6) × Vowel	0.003	0.017	1.003	[0.970, 1.038]	0.204	0.839

Table S3.2 Between-vowel comparisons at each step for RT (log scale; Holm-adjusted)

Step	Contrast	Estimate (log)	SE	Time Ratio	95% CI	z	p
CV_0	i – a	0.083	0.045	1.086	[0.995, 1.186]	1.849	0.064
CV_1	i – a	0.101	0.045	1.106	[1.013, 1.207]	2.251	0.024
CV_2	i – a	0.152	0.045	1.164	[1.066, 1.271]	3.379	0.001
CV_3	i – a	0.123	0.045	1.131	[1.036, 1.235]	2.744	0.006
CV_4	i – a	−0.033	0.045	0.968	[0.886, 1.056]	−0.733	0.464
CV_5	i – a	−0.015	0.045	0.985	[0.903, 1.076]	−0.327	0.744
CV_6	i – a	−0.008	0.045	0.992	[0.909, 1.083]	−0.172	0.864

Table S3.3 Adjacent-step RT contrasts by Age level

Vowel	Contrast	Estimate (log)	SE	Time Ratio	95% CI	z	p
i	CV_1–CV_0	0.023	0.024	1.023	[0.960, 1.090]	0.952	1.000
i	CV_2–CV_1	0.023	0.024	1.024	[0.960, 1.092]	0.963	1.000
i	CV_3–CV_2	−0.008	0.025	0.992	[0.930, 1.059]	−0.312	1.000
i	CV_4–CV_3	−0.126	0.024	0.882	[0.827, 0.940]	−5.195	<.001
i	CV_5–CV_4	−0.012	0.024	0.988	[0.928, 1.052]	−0.497	1.000
i	CV_6–CV_5	−0.007	0.024	0.993	[0.933, 1.058]	−0.291	1.000
a	CV_1–CV_0	0.005	0.025	1.005	[0.942, 1.072]	0.198	1.000

a	CV_2–CV_1	−0.028	0.025	0.973	[0.912, 1.038]	−1.128	1.000
a	CV_3–CV_2	0.021	0.024	1.021	[0.957, 1.089]	0.853	1.000
a	CV_4–CV_3	0.030	0.025	1.030	[0.966, 1.099]	1.221	1.000
a	CV_5–CV_4	−0.030	0.025	0.970	[0.909, 1.036]	−1.215	1.000
a	CV_6–CV_5	−0.014	0.025	0.986	[0.924, 1.052]	−0.564	1.000

S4. Summary of GLMM Analysis of AX Discrimination Accuracy

Table S4.1 Fixed effects (logit estimates and odds ratios with 95% CIs)

Term	Estimate (logit)	SE	Odds Ratio	95% CI	p
(Intercept)	−1.148	0.163	0.317	[0.230, 0.437]	<.001
Vowel (/i/ vs. /a/)	0.415	0.097	1.514	[1.250, 1.832]	<.001
Step 2–1	−0.467	0.123	0.627	[0.493, 0.798]	<.001
Step 3–2	−0.286	0.126	0.751	[0.587, 0.961]	.023
Step 4–3	−0.235	0.128	0.790	[0.615, 1.016]	.066
Step 5–4	−0.342	0.136	0.711	[0.544, 0.928]	.012
Age	−0.200	0.154	0.818	[0.605, 1.107]	.194
Education	0.172	0.153	1.188	[0.881, 1.603]	.259
Vowel × Step 2–1	0.098	0.122	1.103	[0.868, 1.402]	.422
Vowel × Step 3–2	−0.407	0.125	0.665	[0.521, 0.850]	.001
Vowel × Step 4–3	−0.323	0.127	0.724	[0.565, 0.928]	.011
Vowel × Step 5–4	−0.313	0.135	0.731	[0.561, 0.953]	.020
Step 2–1 × Age	0.038	0.121	1.039	[0.820, 1.316]	.751
Step 3–2 × Age	0.148	0.125	1.160	[0.908, 1.481]	.235
Step 4–3 × Age	0.113	0.129	1.120	[0.869, 1.442]	.382
Step 5–4 × Age	−0.301	0.138	0.740	[0.564, 0.970]	.029
Step 2–1 × Education	0.253	0.127	1.288	[1.005, 1.652]	.046
Step 3–2 × Education	0.163	0.131	1.177	[0.909, 1.522]	.216
Step 4–3 × Education	−0.123	0.134	0.884	[0.679, 1.151]	.359
Step 5–4 × Education	−0.082	0.146	0.921	[0.691, 1.227]	.574

Table S4.2 Mean "different" accuracy by vowel (probability scale)

vowel	prob	SE	LCL	UCL
i	0.324	0.035	0.260	0.395
a	0.173	0.031	0.120	0.243

Table S4.3 Estimated means by midpoint × vowel (probability scale; covariates at mean)

vowel	mid	prob	SE	LCL	UCL
i	1	0.5822211	0.04517730	0.4919683	0.6672837
i	2	0.4908157	0.04623775	0.4014659	0.5807562
i	3	0.3250952	0.04138741	0.2497089	0.4107800
i	4	0.2161031	0.03309282	0.1582375	0.2878924
i	5	0.1253127	0.02321003	0.0864256	0.1782825
a	1	0.2292699	0.04293179	0.1559567	0.3238245
a	2	0.1446335	0.03111337	0.0936175	0.2168005
a	3	0.1602343	0.03349744	0.1048554	0.2371138
a	4	0.1724049	0.03533631	0.1136407	0.2528865
a	5	0.1683580	0.03483132	0.1105740	0.2479218

Table S4.4 Adjacent midpoint contrasts within vowel (Holm-adjusted)

vowel	contrast	OR	SE	z	p
i	mid_f2 / mid_f1	0.6916749	0.10986207	-2.321	0.0203
i	mid_f3 / mid_f2	0.4997175	0.08062635	-4.300	0.0001
i	mid_f4 / mid_f3	0.5723137	0.09815392	-3.254	0.0024
i	mid_f5 / mid_f4	0.5196851	0.10140309	-3.354	0.0024
a	mid_f2 / mid_f1	0.5684234	0.10602986	-3.028	0.0098
a	mid_f3 / mid_f2	1.1284453	0.21705696	0.628	1.0000
a	mid_f4 / mid_f3	1.0917779	0.20569030	0.466	1.0000
a	mid_f5 / mid_f4	0.9717753	0.18299780	-0.152	1.0000

Table S4.5 Vowel difference at each midpoint (Holm-adjusted)

mid	contrast	OR	SE	df	z	p
1	i / a	4.684860	1.1389046	Inf	6.353	<.0001
2	i / a	5.700680	1.4300625	Inf	6.939	<.0001
3	i / a	2.524473	0.6328600	Inf	3.694	0.0002
4	i / a	1.323337	0.3371864	Inf	1.100	0.2715
5	i / a	0.707693	0.1895953	Inf	-1.291	0.1969

S5. Summary of LMM Analysis of AX RT

Table S5.1 Fixed effects on log-RT and time ratios (95% CI)

Term	Estimate	SE	Time Ratio	95% CI	z	p
Intercept	-0.715	0.052	0.489	[0.439, 0.544]	-13.66	< .001
Midpoint 2–1	-0.020	0.025	0.981	[0.933, 1.030]	-0.78	0.438
Midpoint 3–2	-0.001	0.025	0.999	[0.951, 1.050]	-0.04	0.968
Midpoint 4–3	0.000	0.025	1.000	[0.952, 1.050]	0.01	0.996
Midpoint 5–4	-0.040	0.025	0.961	[0.915, 1.009]	-1.61	0.108
Vowel (/i/ vs. /a/)	0.082	0.025	1.085	[1.031, 1.143]	3.24	0.003
Age	0.112	0.051	1.118	[1.008, 1.241]	2.19	0.036
L2 Use	-0.111	0.051	0.895	[0.806, 0.993]	-2.18	0.038
Midpoint 2–1 × Vowel	-0.037	0.025	0.964	[0.917, 1.013]	-1.46	0.144
Midpoint 3–2 × Vowel	0.032	0.025	1.032	[0.983, 1.085]	1.27	0.206
Midpoint 4–3 × Vowel	-0.019	0.025	0.981	[0.934, 1.031]	-0.75	0.451
Midpoint 5–4 × Vowel	-0.014	0.025	0.986	[0.939, 1.036]	-0.56	0.577

Table S5.2 Between-vowel RT differences at each midpoint (Holm-adjusted)

Midpoint	Contrast	Estimate	SE	Time Ratio	95% CI	z	p
1	i − a	0.205	0.060	1.228	[1.092, 1.380]	3.44	0.001
2	i − a	0.131	0.060	1.140	[1.014, 1.282]	2.19	0.028
3	i − a	0.195	0.060	1.215	[1.081, 1.366]	3.27	0.001
4	i − a	0.157	0.060	1.170	[1.041, 1.315]	2.64	0.008
5	i − a	0.129	0.060	1.138	[1.013, 1.279]	2.17	0.030

Table S5.3 Adjacent-midpoint RT changes by moderator level

Age

Vowel	Contrast	Time Ratio	95% CI	z	p
i	Midpoint 2–1	0.945	[0.866, 1.032]	-1.60	0.436
i	Midpoint 3–2	1.031	[0.945, 1.126]	0.88	0.759
i	Midpoint 4–3	0.981	[0.899, 1.071]	-0.54	0.759
i	Midpoint 5–4	0.947	[0.868, 1.034]	-1.55	0.436
a	Midpoint 2–1	1.017	[0.930, 1.114]	0.48	1.000
a	Midpoint 3–2	0.968	[0.884, 1.059]	-0.91	1.000
a	Midpoint 4–3	1.019	[0.932, 1.115]	0.53	1.000
a	Midpoint 5–4	0.974	[0.891, 1.065]	-0.73	1.000

L2 Use

Vowel	Contrast	Time Ratio	95% CI	z	p
i	Midpoint 2–1	0.945	[0.866, 1.032]	-1.60	0.436
i	Midpoint 3–2	1.031	[0.945, 1.126]	0.88	0.759
i	Midpoint 4–3	0.981	[0.899, 1.071]	-0.54	0.759
i	Midpoint 5–4	0.947	[0.868, 1.034]	-1.55	0.436

a	Midpoint 2–1	1.017	[0.930, 1.114]	0.48	1.000
a	Midpoint 3–2	0.968	[0.884, 1.059]	-0.91	1.000
a	Midpoint 4–3	1.019	[0.932, 1.115]	0.53	1.000
a	Midpoint 5–4	0.974	[0.891, 1.065]	-0.73	1.000
